# Web Search Queries Can Predict Stock Market Volumes

**DOI:** 10.1371/journal.pone.0040014

**Published:** 2012-07-19

**Authors:** Ilaria Bordino, Stefano Battiston, Guido Caldarelli, Matthieu Cristelli, Antti Ukkonen, Ingmar Weber

**Affiliations:** 1 Yahoo! Research, Barcelona, Spain; 2 ETH Chair of System Design, Zurich, Switzerland; 3 Institute of Complex Systems CNR, “Sapienza” University, Rome, Italy; 4 London Institute for Mathematical Sciences, London, United Kingdom; 5 IMT - Institute for Advanced Studies, Lucca, Italy; Universidad Veracruzana, Mexico

## Abstract

We live in a computerized and networked society where many of our actions leave a digital trace and affect other people’s actions. This has lead to the emergence of a new data-driven research field: mathematical methods of computer science, statistical physics and sociometry provide insights on a wide range of disciplines ranging from social science to human mobility. A recent important discovery is that search engine traffic (i.e., the number of requests submitted by users to search engines on the www) can be used to track and, in some cases, to anticipate the dynamics of social phenomena. Successful examples include unemployment levels, car and home sales, and epidemics spreading. Few recent works applied this approach to stock prices and market sentiment. However, it remains unclear if trends in financial markets can be anticipated by the collective wisdom of on-line users on the web. Here we show that daily trading volumes of stocks traded in NASDAQ-100 are correlated with daily volumes of queries related to the same stocks. In particular, query volumes anticipate in many cases peaks of trading by one day or more. Our analysis is carried out on a unique dataset of queries, submitted to an important web search engine, which enable us to investigate also the user behavior. We show that the query volume dynamics emerges from the collective but seemingly uncoordinated activity of many users. These findings contribute to the debate on the identification of early warnings of financial systemic risk, based on the activity of users of the www.

## Introduction

Nowadays many of our activities leave a digital trace: credit card transactions, web activities, e-commerce, mobile-phones, GPS navigators, etc. This networked reality has favored the emergence of a new data-driven research field where mathematical methods of computer science [1], statistical physics [2] and sociometry provide effective insights on a wide range of disciplines like [3] social sciences [4], human mobility [5], etc.

**Figure 1:**
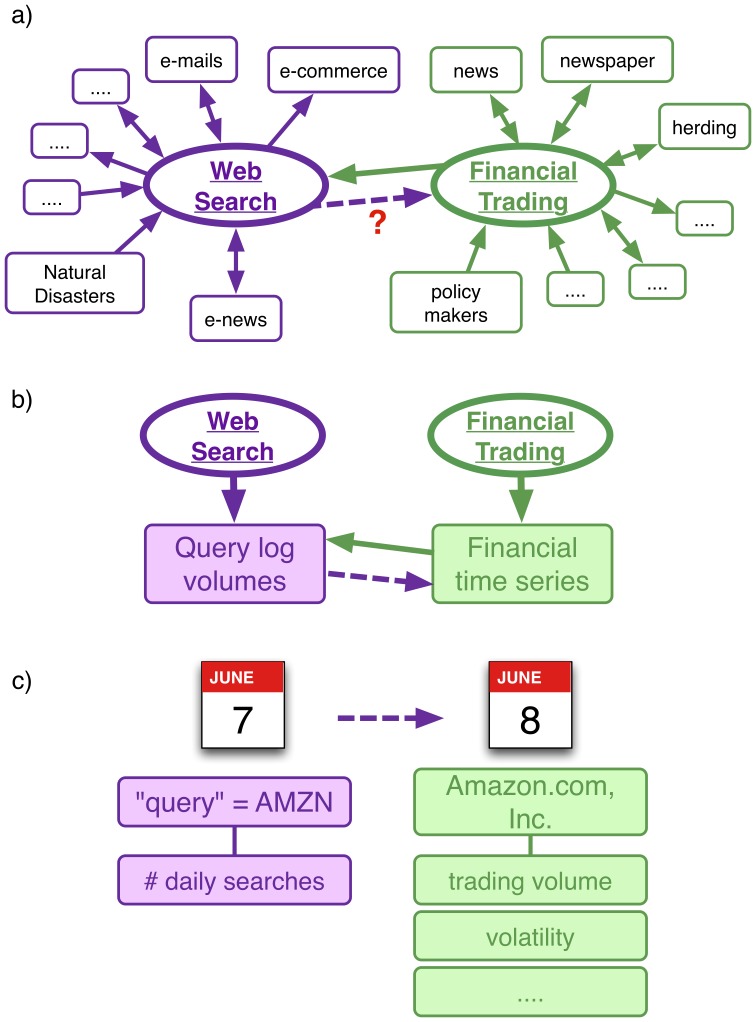
Graphical illustration of the analysis presented in this paper. The study of queries is gaining more and more attention as an important tool for the understanding of social and financial systems. Users perform web searches in order to collect news or browse e-newspaper sites. In particular local or global events such as natural disasters can generate local or global waves of searches through the web. As a result, the logs of these search-engines’ queries are an unprecedented source of anonymized information about human activities. In this paper we provide a detailed analysis on a particular application of these ideas; that is, the anticipation of market activity from user queries. This picture graphically summarizes our procedure. In particular, we investigate which is the relationship between web searches and market movements and whether web searches *anticipate* market activity. While we can expect that large fluctuations in markets, produce spreading of news or rumors or government’s actions and therefore induce web searches (solid green arrow in panel 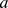), we would like to check if web searches affect or even anticipate financial activity (broken violet arrow in panel 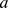). In detail we investigate if today’s query volumes about financial stocks somehow anticipate financial indicators of tomorrow such as trading volumes, daily returns, volatility, etc, (panels 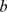 and 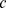) and we find a significant anticipation for trading volumes.

Recent investigations showed that Web search traffic can be used to accurately track several social phenomena [6]–[9]. One of the most successful results in this direction, concerns the epidemic spreading of influenza virus among people in the USA. It has been shown that the activity of people querying search engines for keywords related to influenza and its treatment allows to anticipate the actual spreading as measured by official data on contagion collected by Health Care Agencies [10]. In this paper, we address the issue whether a similar approach can be applied to obtain early indications of movements in the financial markets [11]–[13] (see Fig. 1 for a graphical representation of this issue). Indeed, financial turnovers, financial contagion and, ultimately, crises, are often originated by collective phenomena such as herding among investors (or, in extreme cases, panic) which signal the intrinsic complexity of the financial system [14]. Therefore, the possibility to anticipate anomalous collective behavior of investors is of great interest to policy makers [15]–[17] because it may allow for a more prompt intervention, when this is appropriate. For instance the authors of [18] predict economical outcomes starting from social data, however, these predictions are not in the context of financial markets.

Furthermore it has been shown how volume shifts can be correlated with price movements [19]–[21].

Here, we focus on queries submitted to the Yahoo! search engine that are related to companies listed on the NASDAQ stock exchange. Our analysis is twofold. On the one hand, we assess the relation over time between the daily number of queries (“query volume”, hereafter) related to a particular stock and the amount of daily exchanges over the same stock (“trading volume” hereafter). We do so by means not only of a time-lagged cross-correlation analysis, but also by means of the Granger-causality test. On the other hand, our unique data set allows us to analyze the search activity of individual users in order to provide insights into the emergence of their collective behavior.

## Results

In our analysis we consider a set of companies (“NASDAQ-100 set” hereafter) that consists of the companies included in the NASDAQ-100 stock market index (the 100 largest non-financial companies traded on NASDAQ). We list these companies in Table 1. Previous studies [12] looked at stock prices at a weekly time resolution and found that the volume of queries is correlated with the volume of transactions for all stocks in the S&P 500 set for a time lag of 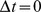 week, i.e. the present week query volumes of companies in the S&P 500 are significantly correlated with present week trading volumes of the S&P 500. In addition, differently from [12] we use daily data from Yahoo! search engine and we look at query volumes from single stocks and do not aggregate these volumes. The authors of [12] suggest that the query volume can be interpreted as reflecting the attractiveness of trading a stock. Further, they find that this attractiveness effect lasts for several weeks and, citing the authors of [12], *present price movements seem to influence the search volume in the following weeks* pointing out that new analysis on data at a smaller time scale are needed.

**Table 1:** The 100 traded companies included in the NASDAQ-100 index with their relative ticker.

Activision Blizzard (ATVI)	Adobe Systems Incorporated (ADBE)	Akamai Technologies, Inc (AKAM)
Altera Corporation (ALTR)	Amazon.com, Inc. (AMZN)	Amgen Inc. (AMGN)
Apollo Group, Inc. (APOL)	Apple Inc. (AAPL)	Applied Materials, Inc. (AMAT)
Autodesk, Inc. (ADSK)	Automatic Data Processing, Inc. (ADP)	Baidu.com, Inc. (BIDU)
Bed Bath & Beyond Inc. (BBBY)	Biogen Idec, Inc (BIIB)	BMC Software, Inc. (BMC)
Broadcom Corporation (BRCM)	C. H. Robinson Worldwide, Inc. (CHRW)	CA, Inc. (CA)
Celgene Corporation (CELG)	Cephalon, Inc. (CEPH)	Cerner Corporation (CERN)
Check Point Software Technologies Ltd. (CHKP)	Cisco Systems, Inc. (CSCO)	Citrix Systems, Inc. (CTXS)
Cognizant Tech. Solutions Corp. (CTSH)	Comcast Corporation (CMCSA)	Costco Wholesale Corporation (COST)
Ctrip.com International, Ltd. (CTRP)	Dell Inc. (DELL)	Dentsplay International Inc. (XRAY)
DirecTV (DTV)	Dollar Tree, Inc. (DLTR)	eBay Inc. (EBAY)
Electronic Arts Inc. (ERTS)	Expedia, Inc. (EXPE)	Expeditors Int. of Washington, Inc. (EXPD)
Express Scripts, Inc. (ESRX)	F5 Networks, Inc. (FFIV)	Fastenal Company (FAST)
First Solar, Inc. (FSLR)	Fiserv, Inc. (FISV)	Flextronics International Ltd. (FLEX)
FLIR Systems, Inc. (FLIR)	Garmin Ltd. (GRMN)	Genzyme Corporation (GENZ)
Gilead Sciences, Inc. (GILD)	Google Inc. (GOOG)	Henry Schein, Inc. (HSIC)
Illumina, Inc. (ILMN)	Infosys Technologies (INFY)	Intel Corporation (INTC)
Intuit, Inc. (INTU)	Intuitive Surgical Inc. (ISRG)	Joy Global Inc. (JOYG)
KLA Tencor Corporation (KLAC)	Lam Research Corporation (LRCX)	Liberty Media Corp., Int. Series A (LINTA)
Life Technologies Corporation (LIFE)	Linear Technology Corporation (LLTC)	Marvell Technology Group, Ltd. (MRVL)
Mattel, Inc. (MAT)	Maxim Integrated Products (MXIM)	Microchip Technology Incorporated (MCHP)
Micron Technology, Inc. (MU)	Microsoft Corporation (MSFT)	Millicom International Cellular S.A. (MICC)
Mylan, Inc. (MYL)	NetApp, Inc. (NTAP)	Netflix, Inc. (NFLX)
News Corporation, Ltd. (NWSA)	NII Holdings, Inc. (NIHD)	NVIDIA Corporation (NVDA)
OÕReilly Automotive, Inc. (ORLY)	Oracle Corporation (ORCL)	PACCAR Inc. (PCAR)
Paychex, Inc. (PAYX)	Priceline.com, Incorporated (PCLN)	Qiagen N.V. (QGEN)
QUALCOMM Incorporated (QCOM)	Research in Motion Limited (RIMM)	Ross Stores Inc. (ROST)
SanDisk Corporation (SNDK)	Seagate Technology Holdings (STX)	Sears Holdings Corporation (SHLD)
Sigma-Aldrich Corporation (SIAL)	Staples Inc. (SPLS)	Starbucks Corporation (SBUX)
Stericycle, Inc (SRCL)	Symantec Corporation (SYMC)	Teva Pharmaceutical Industries Ltd. (TEVA)
Urban Outfitters, Inc. (URBN)	VeriSign, Inc. (VRSN)	Vertex Pharmaceuticals (VRTX)
Virgin Media, Inc. (VMED)	Vodafone Group, plc. (VOD)	Warner Chilcott, Ltd. (WCRX)
Whole Foods Market, Inc. (WFMI)	Wynn Resorts Ltd. (WYNN)	Xilinx, Inc. (XLNX)
Yahoo! Inc. (YHOO)		

This last observation is the starting point of the present work. Is it possible to better investigate the relation between search traffic and market activity on a daily time scale? And, even more important, can query volumes anticipate market movements and be a proxy for market activity? In other words in this paper we are addressing the question whether web searches can be a forecasting tool for financial markets and not only a nowcasting one. This is a novel analysis which try to quantify the link and the direction of the link between search traffic and financial activity.

**Figure 2:**
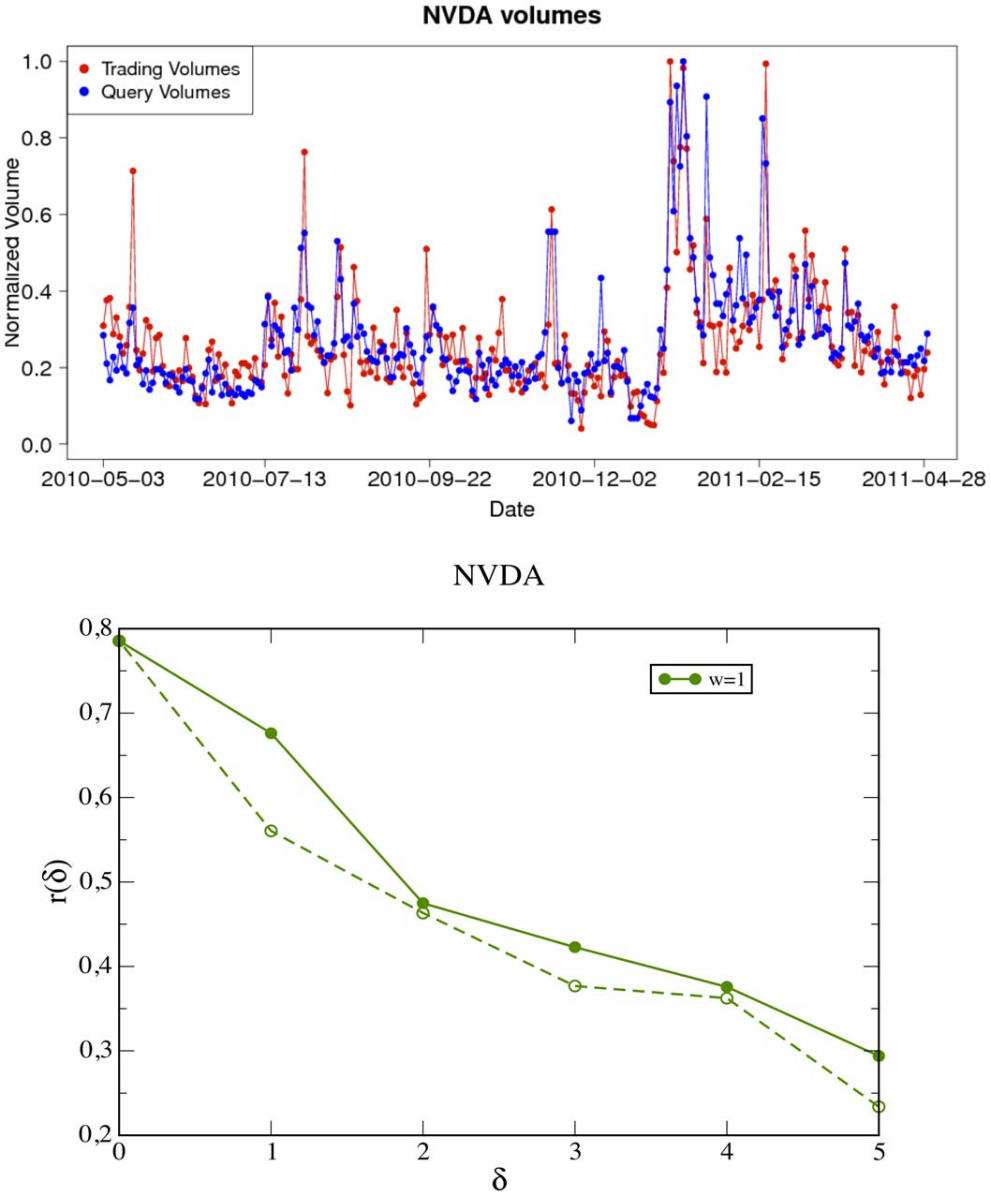
Query log volumes and trading volumes: cross correlation analysis (ticker: “NVDA”). (**up**) Time evolution of normalized query-logs volumes for the ticker “NVDA” compared with the trading-volume of the “NVIDIA Corporation”. The data for both query-logs (blue) and trading volume (red) are aggregated on a daily basis. (**bottom**) The plot of the sample cross correlation function 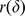 as defined in Eq. (1) 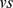 absolute values of the time lag 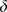 (positive values of 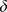 correspond to solid lines while negative values of the time lag correspond to the broken lines). The correlation coefficients at positive time lags are always larger than the corresponding at negative ones, this suggests that today’s query volumes anticipate and affect the trading activity of the following days (typically one or two days at most).

**Figure 3:**
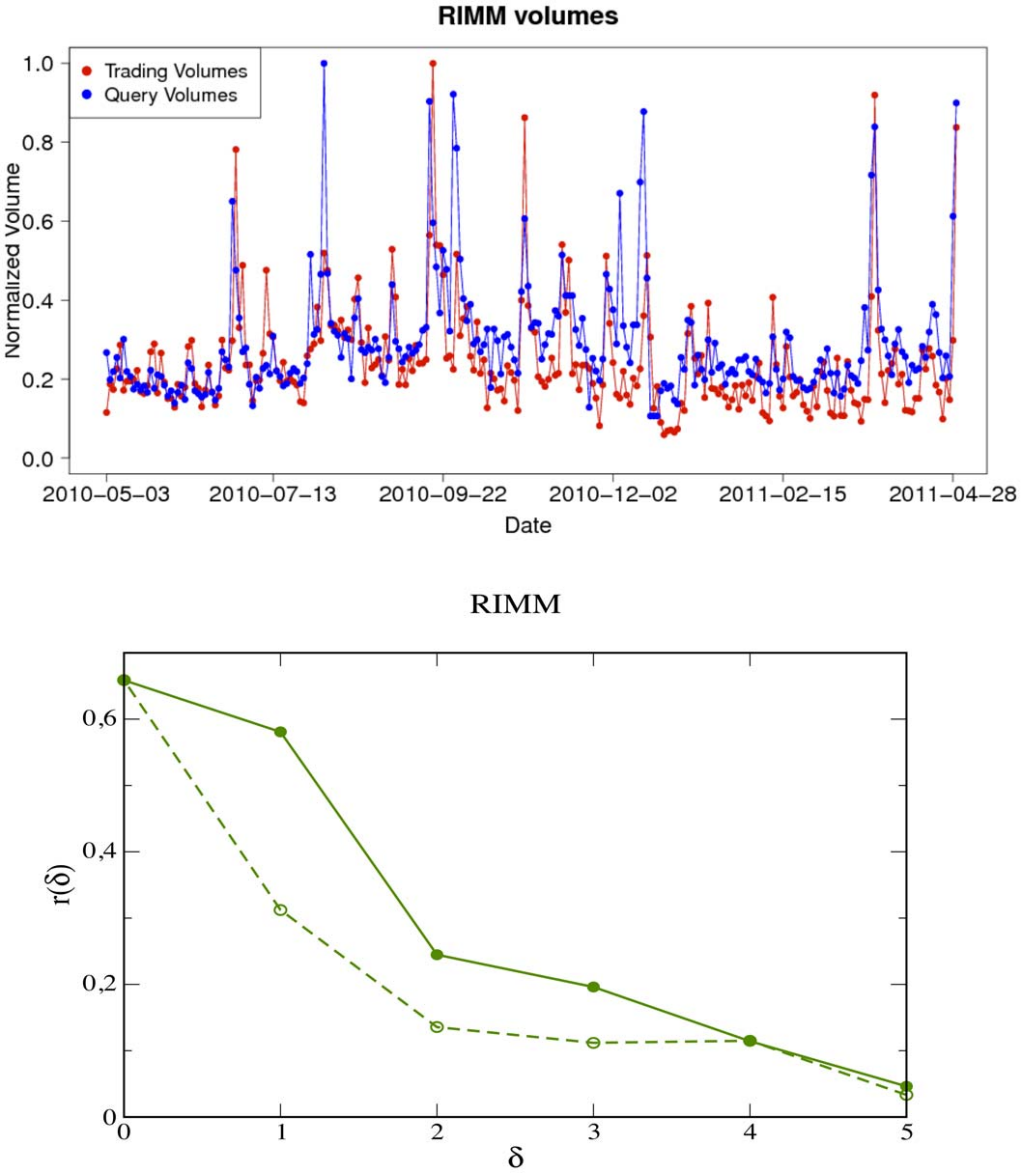
Query log volumes and trading volumes: cross correlation analysis (ticker: “RIMM”). (**up**) Time evolution of normalized query-logs volumes for the ticker “RIMM” compared with the trading-volume of the “Research In Motion Limited”. The data for both query-logs (blue) and trading volume (red) are aggregated on a daily basis. (**bottom**) The plot of the sample cross correlation function 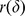 as defined in Eq. (1) vs absolute values of the time lag 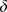 (positive values of 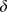 correspond to solid lines while negative values of the time lag correspond to the broken lines). As in the case of the ticker “NVDA” corresponding to the company “NVIDIA Corporation” in Fig. 2, the correlation coefficients at positive time lags are always larger than the corresponding at negative ones, this suggests that today’s query volumes anticipate and affect the trading activity of the following days (typically one or two days at most).

**Figure 4:**
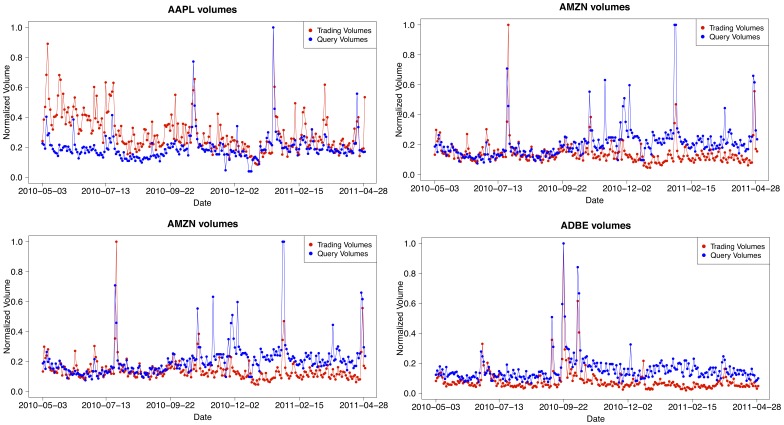
Query volumes and trading volumes. We plot the query-search volumes and trading volumes time series for four stocks (AAPL, AMZN, NFLX and ADBE) to show that the patterns observed in Figs. 2 and 3 are common to most of stocks of the set considered (NASDAQ-100).

We consider search traffic as well as market activity at a daily frequency and find a strong correlation between query volumes and trading volumes for all stocks in the NASDAQ-100 set. Fig. 2 (top panel) shows the time evolution of the query volume of the ticker “NVDA” and the trading volume of the corresponding company stock “NVIDIA Corporation” and Fig. 3 (top panel) shows the same plot for query volume of the ticker “RIMM” and the trading volume of the company stock “Research In Motion Limited” (see also Section “Materials and Methods”). A simple visual inspection of these figures (see also Fig. 4) reveals a clear correlation between the two time series because peaks in one time series tend to occur close to peaks in the other.

The lower panels of Figs. 2 and 3 report the values of cross correlation between trading and query volume as a function of the time lag 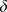 defined as the time-lagged Pearson cross correlation 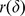 coefficient between two time series 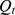 and 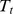:
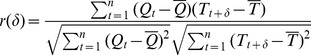
(1)where 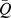, 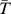 are the sample averages of the two time series (in this case 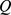 and 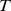 represent query and trading volumes, respectively). The coefficient 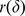 can range from 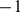 (anticorrelation) to 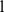 (correlation).

The cross correlation coefficients for positive values of 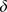 (solid lines) are always larger than the ones for negative time lag (broken lines). This means that query volumes tend to anticipate trading volumes. Such an anticipation spans from 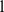 to 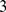 days at most.

Beyond a lag of 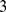 days, the correlation of query volumes with trading volumes vanishes. In Table 2 where we report the cross correlation function between queries and trading volumes averaged over the 87 companies in the NASDAQ-100 for which we have a clean query-log signal (see also Tables 3, 4 and 5 where we find similar results for a different definition of query volumes and for all the stocks from NASDAQ-100 without any filtering procedure, these results are extensively discussed in Section “Materials and Methods”). In Table 6 instead we report the cross correlation functions for some of the 87 companies investigated in Table 2 (for the sake of completeness in Supporting Information S1 we report the tables of cross correlation functions for all the clean stocks and the cross correlation functions for those stocks characterized by spurious origin of the query volume).

**Table 2:** Average cross-correlation functions for the clean NASDAQ-100 stocks (query: Ticker, volumes: searches).

*δ*	−5	−4	−3	−2	−1	0	1	2	3	4	5
CCF	0.0176	0.0604	0.0657	0.0993	0.1816	0.3641	0.2700	0.1145	0.0834	0.0540	0.0312

By clean stocks we mean that we remove those stocks which give rise to spurious queries such as the one containing a common words like LIFE or for instance the stock EBAY. In Supporting Information S1 we report the cross correlation functions of the 87 stocks on which the average is performed.

**Table 3:** Average cross-correlation time series for NASDAQ-100 clean stocks (query: Ticker, volumes: users).

*δ*	−5	−4	−3	−2	−1	0	1	2	3	4	5
CCF	0.0078	0.0344	0.0501	0.0736	0.1482	0.3194	0.2349	0.0876	0.0623	0.0345	0.0151

The results from the queries of Yahoo! users or from all searches (Table 2) are almost identical.

**Table 4:** Average cross-correlation time series for NASDAQ-100 stocks (query: Ticker, volumes: searches).

*δ*	−5	−4	−3	−2	−1	0	1	2	3	4	5
CCF	0.0067	0.0487	0.0507	0.0806	0.1510	0.3150	0.2367	0.0940	0.0675	0.0433	0.0197

**Table 5:** Average cross-correlation time series for NASDAQ-100 stocks (query: Company name, volumes: searches).

*δ*	−5	−4	−3	−2	−1	0	1	2	3	4	5
CCF	0.0159	0.0629	0.0508	0.0455	0.0639	0.1196	0.1083	0.0561	0.0509	0.0299	0.0169

Correlations are lower than the case in which we consider the queries deriving from the tickers (Table 4).

**Table 6:** Values of cross-correlation functions for some selected stocks.

Ticker	*δ = *−5	*δ = *−4	*δ = *−3	*δ = *−2	*δ = *−1	*δ = *0	*δ = *1	*δ = *2	*δ = *3	*δ = *4	*δ = *5
ADBE	0.08	0.12	0.14	0.19	0.47	0.83	0.51	0.19	0.09	0.10	0.11
CEPH	0.16	0.26	0.22	0.14	0.32	0.80	0.44	0.24	0.12	0.13	0.15
APOL	0.02	0.06	0.10	0.21	0.43	0.79	0.55	0.22	0.12	0.07	0.03
NVDA	0.23	0.36	0.38	0.46	0.56	0.79	0.68	0.47	0.42	0.38	0.29
CSCO	0.04	0.07	0.13	0.36	0.53	0.74	0.63	0.34	0.26	0.17	0.12
AKAM	−0.04	−0.06	0.03	0.07	0.22	0.72	0.49	0.20	0.11	0.02	-0.01
NFLX	0.10	0.16	0.16	0.24	0.47	0.68	0.54	0.25	0.19	0.16	0.13
ISRG	0.07	0.13	0.18	0.21	0.38	0.67	0.64	0.29	0.20	0.11	0.05
RIMM	0.03	0.12	0.11	0.14	0.31	0.66	0.58	0.24	0.20	0.11	0.05
FFIV	0.06	0.06	0.13	0.21	0.35	0.65	0.56	0.33	0.21	0.14	0.13

The values of the cross-correlation function 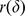 for 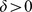 is always higher than the value of 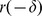. From this evidence it appears that query volumes anticipate trading volumes by one or two days. See Supporting Information S1 for the complete results for the 87 clean stocks.

As a first result from this analysis we find that the significant correlation between query volumes and trading volumes at 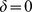 confirms the results of [12] also at a daily timescale. Our findings (i.e. positive correlation for negative time lags) also support the vision that present market activity influences future users’ activity but in contrast with [12] the length of this *influence* appears to be much shorter than what expected (only few days). It appears that the correlation only emerges at a daily scale and seems to be not observed at weekly resolution.

However, the most striking result is that the cross-correlation coefficients between present query volumes and future trading volumes appears to be larger than the coefficient of the opposite case. In the following of this paper we discuss in detail this anticipation effect and give a statistical validation of our finding.

### Statistical Validation

In order to assess the statistical significance of the results for the NASDAQ-100 set, we construct a reshuffled data set in which the query volume time series of a company 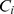 is randomly paired to the trading volume time series of another company 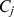. The values of the cross-correlation coefficient averaged over 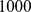 permutations (values which span the range 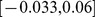) are smaller than the original one (which is 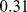) by a factor 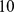. The residual correlation present in the reshuffled dataset can be explained in terms of general trends of the market and of the specific (technological) sector considered [22]–[24].

As a second test we remove the top five (and ten) largest events from the trading volume times series in order to verify if the results shown in Table 6 (the results for all the stocks are reported in Supporting Information S1) are dominated by these events. In Table 7 we report the comparison between the values of the cross correlation coefficient 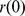 of the two series for a selection of stocks. A significant correlation is still observed for most of the stocks considered. This important test supports the robustness of our findings. In fact, even if the drop indicates that the distributions underlying the investigated series are fat-tailed (see Supporting Information S1and the discussion about the validity of the Granger test in the following of the paper) and that a significant fraction of the correlation is driven by largest events (about 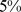 of the events are responsible for 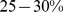 of the correlation on the average), more than half of the correlation (for some stocks this percentage reaches 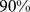) cannot be explained by these extreme events.

**Table 7:** Cross-correlation coefficient 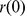 between query and trading volumes after removing largest events.

Ticker	*r*(0)	*r*(0)−Top5	*r*(0)−Top 10
ADBE	0.83	0.51	0.32
CEPH	0.80	0.32	0.24
APOL	0.79	0.55	0.46
NVDA	0.79	0.70	0.64
CSCO	0.74	0.56	0.46
AKAM	0.72	0.51	0.39
NFLX	0.68	0.62	0.62
ISRG	0.67	0.57	0.55
RIMM	0.66	0.59	0.52
FFIV	0.65	0.55	0.50

We compute the cross-correlation coefficient 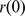 between query and trading volumes after removing the days characterized by the highest trading volumes, respectively the top five and top ten events are removed. We note that a significant correlation is still observed for most of the stocks considered. This important test supports the robustness of our findings. See Supporting Information S1for the complete results for the 87 clean stocks.

**Figure 5:**
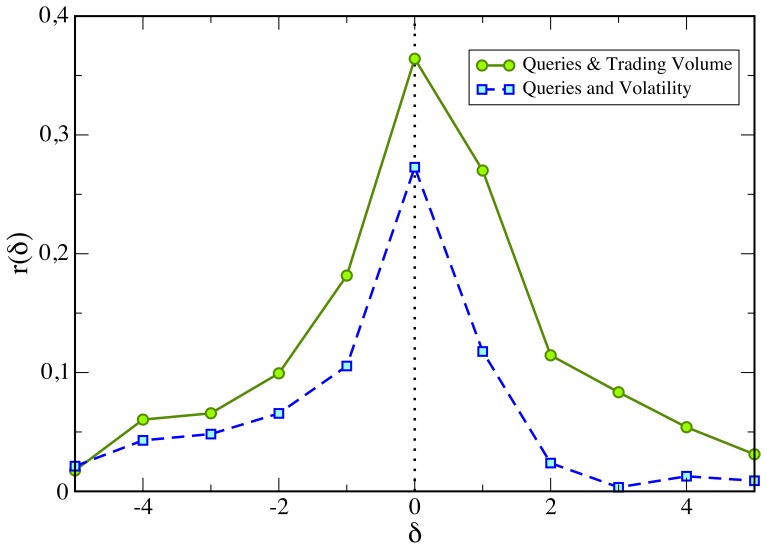
Comparison of the cross-correlation function between query volumes and trading volumes and query volumes and volatility. Trading volume and volatility are correlated and given the fact that volatility is also autocorrelated, the correlation between present query volume and future trading volume could be simply originated by this autocorrelated term. However, we show that the cross-correlation between query and volatility (broken line) is significantly smaller than the one between query and trading volume (solid line). Moreover the 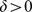 branch in the volatility case is equal or even smaller than the value observed in the 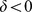 one. If the origin of the effect were due to the autocorrelation component of the volatility, we would expect a similar behavior for both cross-correlation function. This facts support that the non-autocorrelated origin of the correlation between between present query volume and future trading volume. As a proxy for the volatility we use the absolute value of daily price returns.

Turning now the discussion towards the validation of the fact that query volumes anticipate trading volumes, as a first issue, it is a well-known fact that trading volumes and volatility are correlated and this last appears to be autocorrelated [25]–[27] (the decay of the volatility is well-described by a power law with an exponent ranging between 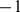 and 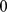). Therefore the correlation between the query volumes and the future trading volumes shown in Figs. 2 and 3 could be explained in terms of these two effects. In this respect we compare the lagged cross-correlation function between a proxy for the volatility (the absolute value of price returns) and the query volumes with the results shown in Table 2. As shown in Fig. 5, the 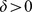 branch in the volatility case is equal or even smaller than the value observed in the 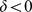 one, differently from the trading volume case. If the origin of the effect were due to the autocorrelation component of the volatility, we would expect a similar behavior for both cross-correlation functions. In addition we observe that the volatility autocorrelation function decays much slower (from weeks to months) than the typical time decay of the cross correlations here investigated (few days). This supports the non-autocorrelated origin of the anticipation effect.

As a second measure of the anticipation effect, we also performed a Granger causality test [28] in order to determine if todays search traffic provides significant information on forecasting trading volumes of tomorrow. We find that trading volumes can be considered Granger-caused by the query volume. We want to point out that Granger-causality does not imply a causality relation between the two series. In fact it can be argued with a simple counterexample that two Granger-caused series may be driven by a third process and therefore the interpretation of the Granger relation as a causality link would be wrong. In our analysis the results of the Granger test are only used to assess the direction of the anticipation between queries and trading activity. In this sense we claim that query volumes observed today are informative of (and consequently forecast) tomorrows trading volumes.

Furthermore, the fat-tailed nature of the distributions under investigation (see Supporting Information S1) may weaken the results of the Granger-test which, in principle, requires gaussian distributions for the error term of the regressions [28]. However, we perform a series of additional analyses and tests which support and confirm the picture coming from Granger-test results (see Section “Materials and Methods” for further details).

### Users’ Behavior

**Figure 6:**
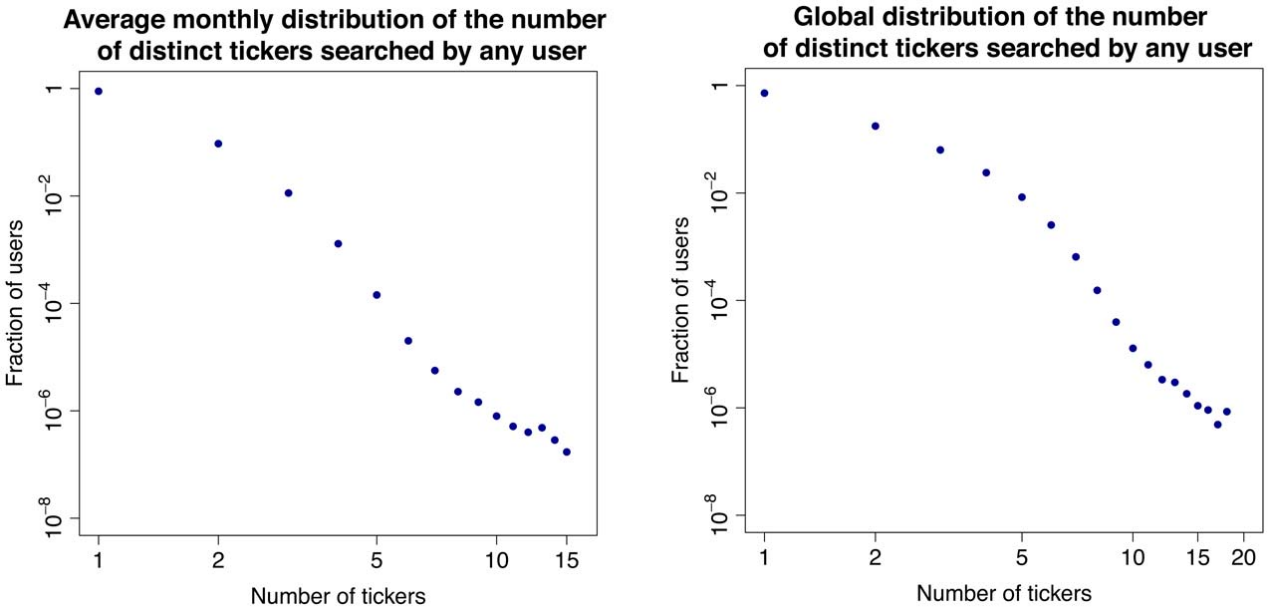
Typical users’ behavior. Average (left) monthly and (right) yearly distribution of the number of distinct tickers searched by any Yahoo! user.

**Figure 7:**
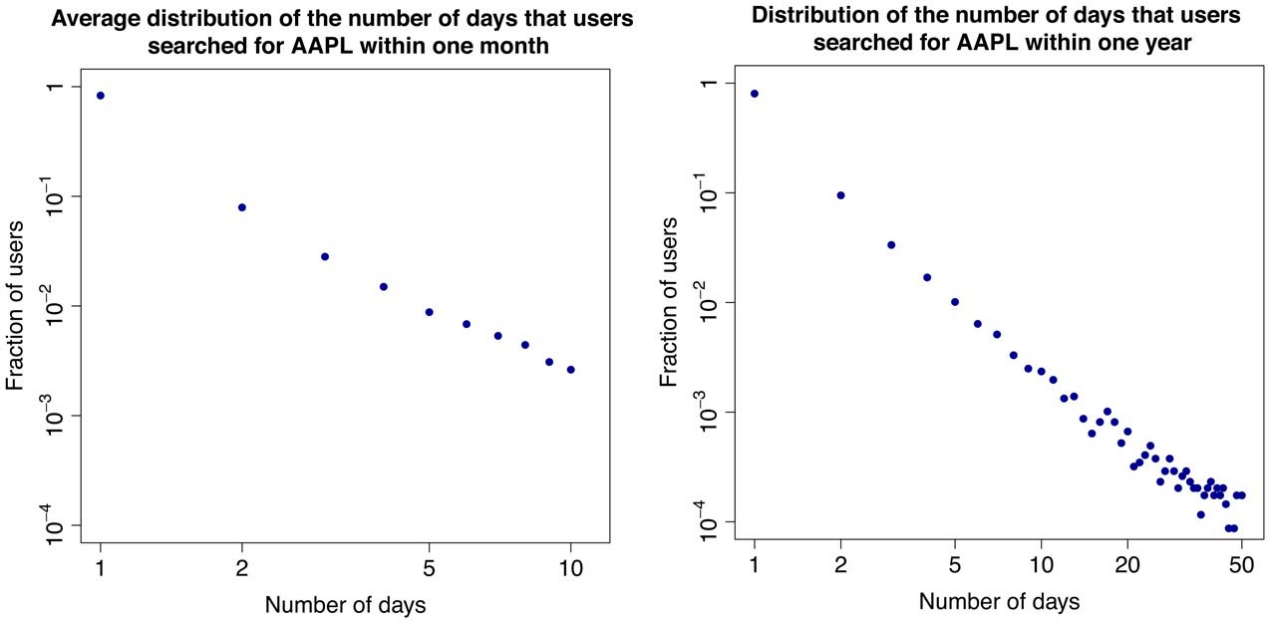
Behavior of the users who search for AAPL. Distribution of the number of days that users searched for AAPL within one month (left) and over the whole year (right).

**Figure 8:**
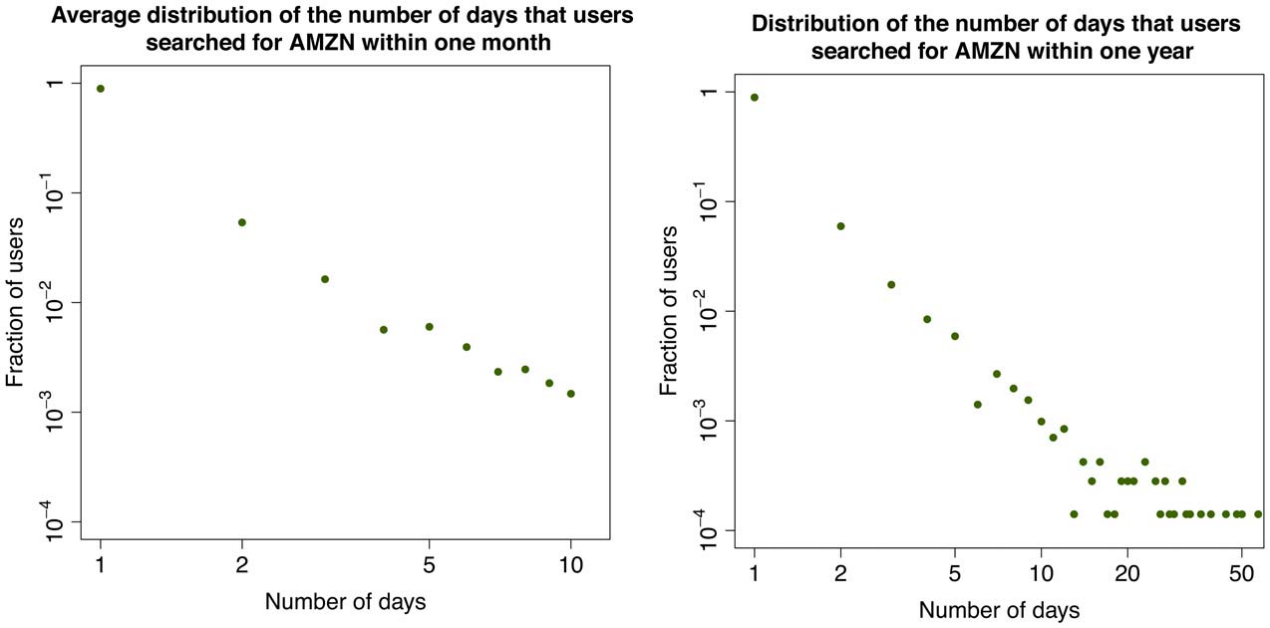
Behavior of the users who search for AMZN. Distribution of the number of days that users searched for AMZN within one month (left) and over the whole year (right).

**Figure 9:**
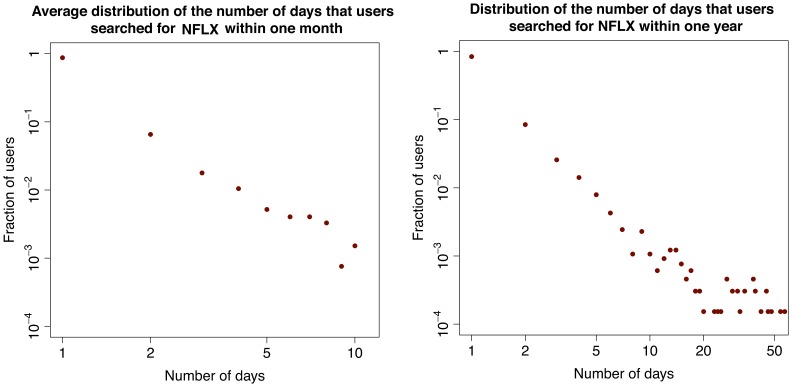
Behavior of the users who search for NFLX. Distribution of the number of days that users searched for NFLX within one month (left) and over the whole year (right).

**Figure 10:**
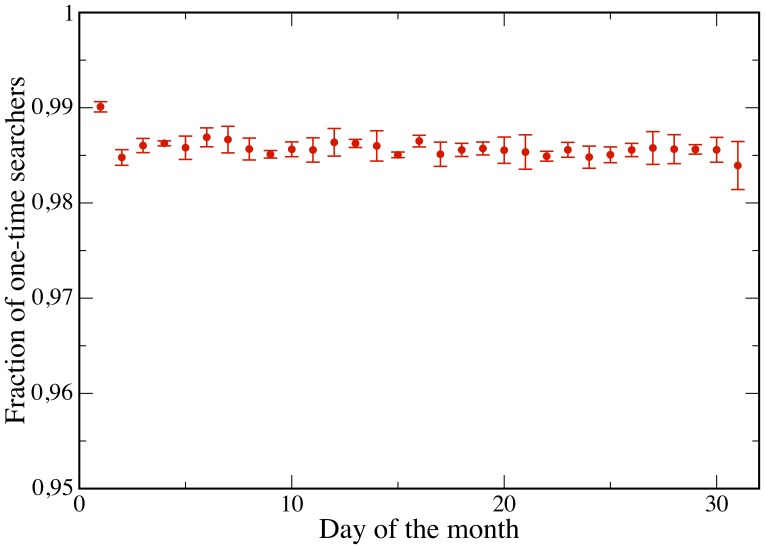
Evolution of the percentage of one-time searchers. The fraction of one-time searchers appear to be very stable in time and we do not observe a correlation of these kind of users with anomalous trading volume or price movements.

In the second part of our investigation we focus on the activity of single users. We are able to track the users who have registered to Yahoo! and thus have a Yahoo! profile. One could expect that users regularly query a set of tickers corresponding to stocks of their interest. This is because for queries that match the ticker of a stock, the search engine shows the user up-to-date market information about the stock in a separate display that appears above the normal search results. In addition, if any important news appears, the corresponding page would show among the top links in the search result. Therefore, we first compute the distribution of the number of tickers searched by each user in various time windows and time resolution (see Fig. 6). Interestingly, most users search only one ticker, not only within a month, but also within the whole year. This result is robust along the time interval under observation and across tickers. As a further step, among the users who search at least once a given ticker in a certain time window, we compute the distribution of the number of different days in which they search again for the same ticker. In this case, we restrict the analysis to some specific tickers, namely to those with highest cross correlation between query volumes and trading volumes (e.g., those for Apple Inc., Amazon.com, Netflix Inc.). Surprisingly, as shown in Section “Materials and Methods” and in Figs. 7, 8, 9, the majority of users (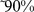) searched the ticker only once, not only during a month, but also within a year. Again, this result is robust along the 12 months in our dataset. Altogether, we find that most users search for one “favorite” stock, only once. The fact that these users do not check regularly a wide portfolio of stocks suggests that they are not financial experts. In addition, there is no consistent pattern over time. Users perform their searches in a seemingly uniform way over the months. In addition we find that our results are typical and very stable in time. In fact in this respect we do not observe any correlation between large fluctuations of trade volume, large price drops and influx of one-time searchers or with large price drops. In Fig. 10 we show the evolution of one-time searchers which appears to be very stable in time.

Overall, combining the evidence on the relation between query and trading volumes with the evidence on individual user behavior, brings about a quite surprising picture: movements in trading volume can be anticipated by volumes of queries submitted by non-expert users, a sort of *wisdom of crowds* effect.

## Discussion

In conclusion, we crawled the information stored in query-logs of the Yahoo! search engine to assess whether signals in querying activity of web users interested in particular stocks can anticipate movements in trading activity of the same stocks. Differently from previous studies we considered daily time series and we focused on trading volumes rather than prices.

Daily volumes of queries related to a stock were compared with the effective trading volume of the same stock by computing time-delayed cross-correlation.

Our results show the existence of a positive correlation between todays stock-related web search traffic and the trading volume of the same stocks in the following days. The direction of the correlation is confirmed by several statistical tests.

Furthermore, the analysis of individual users’ behavior shows that most of the users query only one stock and only once in a month. This seems to suggest that movements in the market are anticipated by a sort of “wisdom of crowd” [29]. These findings do not explain the origin of the market movements but shows that that search traffic can be a good proxy for them.

Furthermore, if one could assume that queries of a user reflect the composition of her investment portfolio, our finding would suggest that most of the investors place their investments in only one or two financial instruments. The assumption that queries reflect portfolio composition is a strong hypothesis and cannot be verified in our data at the current stage. The finding would then deviate from the diversification strategy of the well-known Markovitz approach, but would be in line with previous empirical works on carried out on specific financial markets. This result, if confirmed, could have very important consequences. In epidemics, by taking for granted that everybody has a mean number of contacts brings to incorrect results on disease propagations. Here the assumption that investors portfolio is balanced, while it is not, could explain why domino effects in the market are faster and more frequent than expected.

This does not mean that we can straightforwardly apply the models of epidemic spreading [30]–[32] to financial markets. In fact, in the latter case (differently from ordinary diseases) panic spreads mostly by news. In an ideal market, all the financial agents can become “affected” at the same time by the same piece of information. This fundamental difference makes the typical time scale of reactions in financial markets much shorter than the one in disease spreading. It is exactly for that reason that any early sign of market behavior must be considered carefully in order to promptly take the necessary countermeasures. We think that this information can be effectively used in order to detect early signs of financial distress.

We also believe this field to be very promising and we are currently working on the extension of this kind of web analysis to twitter data and semantic analysis of blogs.

## Materials and Methods

In this section we give a detailed overview of the investigations carried out in this paper. The first contribution of our work consists, as previously said, of an analysis of the relation between the activity of the users of the Yahoo! search engine and real events taking place within the stock market. Our basic assumption is that any market activity in an individual stock may find some correspondence in the search activity of the users interested in that stock. Thus we study whether significant variations in the stock trading volumes are anticipated by analogous variations in the volume of related Web searches. To investigate the existence of a correlation between query volumes and trading volumes, we compute time-lagged *cross-correlation coefficients* of these two series.

We conduct such analysis performing separate experiments to test the two different query definitions that we take into consideration, i.e., queries containing the stock ticker string, or queries matching the company name. The results of this first set of experiments are presented in Subsection “Correlation between query volumes and trading volumes”.

We then apply permutation tests, Granger-causality test and several analyses to assess the significance of the correlations found. These experiments are described in Subsection “Statistical validation of query anticipation”.

Finally, Subsection “Analysis of users’ behavior” presents details of the last part of our work, where we try to gain a better knowledge of the typical behavior of the users who issue queries related to finance. Here we refine our analysis of the information extracted from query logs to understand what a typical user searches for, such as whether she looks for many different tickers or just for a few ones, and, if she looks for them regularly or just sporadically.

### Database

#### The stocks analyzed

In this work we compare query volumes and trading volumes of a set of companies traded in the NASDAQ (National Association of Securities Dealers Automated Quotation) stock exchange, which is the largest electronic screen-based equity securities trading market in the United States and second-largest by market capitalization in the world. Precisely, we analyze the 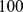 companies included in the NASDAQ-100 stock-market capitalization index. These companies are amongst the largest non-financial companies that are listed on the NASDAQ (technically the NASDAQ-100 is a modified capitalization-weighted index, it does not contain financial companies and it also includes companies incorporated outside the United States.) We list these companies in Table 1. The daily financial data for all of stocks is publicly available from Yahoo! Finance (see http://finance.yahoo.com/ *)* and we focus our attention on the daily trading volumes.

#### Query data

The query-log data we analyze is a segment of the Yahoo! US search-engine log, spanning a time interval of one year, from mid-2010, to mid-2011. The query-log stores information about actions performed by users during their interactions with the search engine, including the queries they submitted and the result pages they were returned, as well as the specific documents they decided to click on.

We compute query volume time series by extracting and aggregating on a daily basis two different types of queries for each traded company:

- all queries whose text contains the stock ticker string (i.e. “YHOO” for Yahoo!) as a distinct word;
- all queries whose text exactly matches the company name (after removing the legal ending, “Incorporated” or “Corporation” or “Limited”, and all their possible abbreviations).

All queries in the log are associated with a timestamp that represents the exact moment the query was issued to the search engine. We use this temporal information to aggregate the query volumes at different levels of granularity. Furthermore, every action is also annotated with a cookie, representing the user who submitted the query. These cookies allow to track the activity of a single user during a time window of a month. By using this information, we also computed *user volumes* by counting the daily number of distinct users who made at least one search related to one company (according to the query definitions provided above). Thus, for each stock taken into consideration, we can compare the daily volumes of related queries, as well as the number of distinct users issuing such queries per day with the daily trading volumes gathered from Yahoo! Finance.

### Correlation between Query Volumes and Trading Volumes

We compare the query volume of every stock with the trading volume of the same stock. The two definitions of queries introduced are used in separate experiments, that is, in one case we aggregate all the queries containing the ticker of a company, and in another case we only consider queries that match the company name.

**Figure 11:**
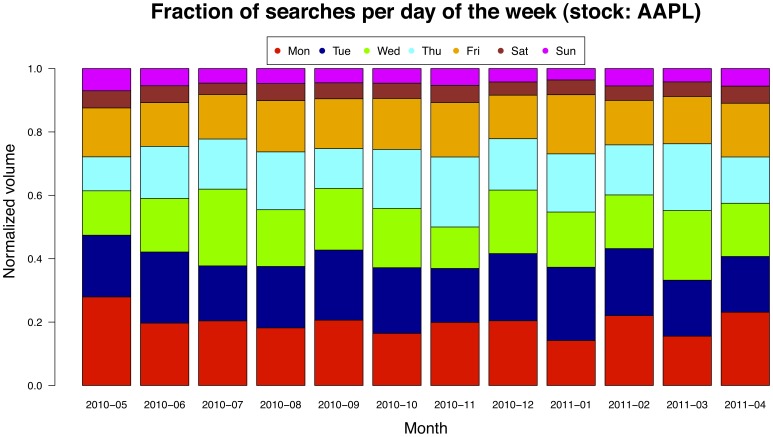
Query-search for AAPL stock in the various days of the week. Query volumes of NASDAQ-100 tickers are negligible during non-working days, then we consider only the contribution to query volumes deriving from working days.

We extract from both data sources (the query volumes and the trading volumes of a given stock) a time series composed by daily values in the time interval ranging from mid 2010 to mid 2011. Although the query-log contains information collected during holidays and weekends as shown in Fig. 11 for the case of the AAPL stock, the financial information is obviously only available for trading days. Thus, for the sake of uniformity, we filter out all the non-working days from the query volume time series. In the end, we obtain two time series of 250 working days for every stock.

As a second step, given the time series 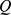 of the query volumes and the time series 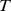 of trading volumes, we compute the *cross-correlation coefficient* 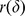 for every company.

This correlation coefficient ranges from 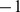 to 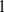. Although the above coefficient can be computed for all delays 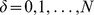, we chose to consider a maximum lag of one week (five working days).

Tables 4 and 5 report the results obtained for these experiments. Columns instead correspond to different values of the time-lag 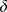 used in the calculation of the cross-correlation coefficients. We observe that the cross-correlation coefficients always assume nearly equal to zero for 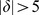.

**Table 8:** Average cross-correlation functions between search-engine volumes and signed price returns for the clean NASDAQ-100 stocks (query: Ticker, 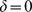).

Volume	Price returns	Avg correlation
searches	*P*_+_	0.2650
searches	*P*_−_	−0.2360
searches	*P*_*A*_	0.2728
users	*P*_+_	0.2722
users	*P*_−_	−0.1975
users	*P*_*A*_	0.2446

When the first query definition is taken into consideration (ticker query), the average cross-correlation coefficient in the base case of 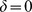 is equal to 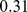. Similar values are obtained if a time-lag 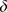 in the range 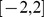 is considered. It is worth noticing that for some individual companies we observe much higher correlations. On this account Table 6 presents the best results for single stocks (see Supporting Information S1for the complete results: it is worth noticing that considering only the stocks for which 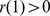, there are 8 stocks for which 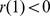, for 68 stocks it holds that 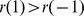 while for the remaining 11 stocks we observe 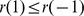). For these companies, we also report in Table 7 (see Supporting Information S1for all the results) the basic cross-correlation at lag 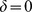 after removing from the time series the days corresponding to the top 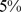 and 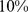 values of the trading volume. It is interesting to observe that the correlations are still significant and so the correlation does not seem to be due only to peak events, which generally correspond to headlines in the news, product announcements or dividend payments.

When the second query definition (company names) is considered, we observe weaker correlations than the previous case. The average cross-correlation coefficient in the base case 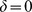 is equal to 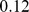.

In addition we point out that the process of extracting data from query-logs can introduce spurious queries which have a non financial origin. Especially some of the ticker queries match our above definition, but are nonetheless unrelated to the stock represented by the ticker. For instance, some ticker strings correspond to natural language words, such as “FAST” (Fastenal Company) and “LIFE” (Life Technologies Corp.). As one can reasonably expect, the overwhelming majority of queries containing these words are completely unrelated to the companies that are the subject of our study. Other cases of companies for which we discovered very large levels of noise included e-commerce portals like Ebay. In all these cases the ticker often appears in navigational queries that are unrelated to the company stock (see Supporting Information S1). For this reason, we filter out all companies whose query volumes are discovered to be noisy, retaining a smaller, but cleaner set of 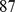 companies for which the spurious queries are a negligible fraction. By restricting the computation of the cross-correlation function to these companies, we observe a larger value of the average cross-correlation. Table 2 reports the results obtained for the first query definition (queries including the ticker as a distinct word), which represents the case for which the best performances of the queries are observed. The average cross-correlation at time lag 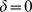 is 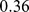.

**Table 9:** Granger causality test.

Dataset	lag(days)	Direction	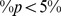	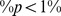	Avg reduction in RSS
Q (100 tickers)	1	Q 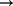 T	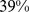	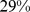	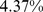
Q (100 tickers)	1	T 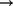 Q	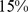	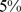	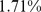
U (100 tickers)	1	U 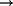 T	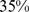	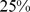	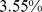
U (100 tickers)	1	T 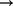 U	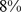	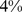	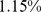
Q (100 tickers)	2	Q 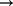 T	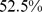	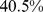	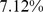
Q (100 tickers)	2	T 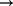 Q	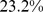	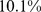	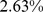
U (100 tickers)	2	U 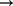 T	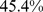	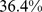	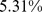
U (100 tickers)	2	T 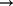 U	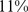	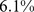	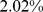
Q (87 tickers)	1	Q 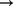 T	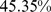	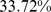	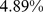
Q (87 tickers)	1	T 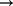 Q	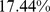	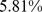	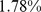
U (87 tickers)	1	U 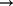 T	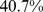	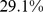	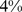
U (87 tickers)	1	T 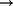 U	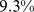	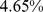	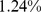
Q (87 tickers)	2	Q 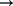 T	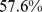	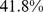	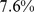
Q (87 tickers)	2	T 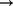 Q	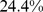	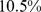	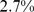
U (87 tickers)	2	U 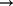 T	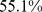	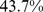	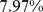
U (87 tickers)	2	T 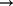 U	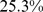	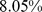	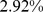

Adding information about yesterday’s query volume reduces the average prediction error (in an autoregressive model) for today’s trade volume by about 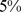, and for half of the companies the reduction is statistically significant at 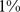.

Besides query volumes, we also consider user volumes, i.e., the number of distinct users who issued queries related to a company in any given day. For reasons listed above, this analysis is restricted to the 87 NASDAQ-100 companies for which we have a clean query-log signal. Cross-correlations between user volumes and trading volumes are shown in Table 3. We observe similar findings to the ones obtained in the previous experiments, although the average cross-correlation is 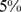 smaller than the one obtained with query volumes. The average cross-correlation between user volumes and trading volumes at time lag 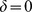 is 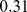.

### Statistical Validation of the Query Anticipation

#### Permutation test

A permutation test, also called randomization test, is a statistical significance test where random rearrangements (or permutations) of the data are used to validate a model. Under the null hypothesis of such a test data permutations have no effect on the outcome, and the reshuffled data present the same properties as the true instance. The rank of the real test statistic among the shuffled test statistics determines the empirical “p-value”, which is the probability that the test statistic would be at least as extreme as observed, if the null hypothesis were true. For example, if the value of the original statistic is 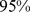 greater than the random values, we can reject the null hypothesis with a confidence 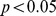. This means that the probability that we would observe a value as extreme as the true one, if the null hypothesis were true, is less than 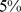. In our setting, the aim is to verify the significance of the correlation between the queries containing the ticker of a company and the trade volumes of the same company. In particular, we want to assess if the cross-correlation between query volume and trading volume of a given company is higher than the cross-correlation between query volume of company 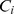 and trading volume of some other company 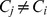. The purpose of this test is to show that the correlations we observe are not merely a consequence of stock market related web search activity being correlated with stock market activity *in general*.

Our original data is given by the set of pairs of time series 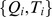 previously considered. Every pair in this set contains information concerning a given company 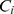. As already indicated, 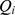 is the time series of the query volumes of 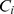, whereas 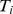 is the time series of the trading volumes of 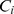. We use as test statistic the cross-correlation coefficient between 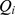 and 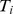. Starting from the above data, we apply 1000 random permutations to create an ensemble of 1000 distinct datasets, each one composed of pairs 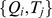, where the time series of query volumes of a company 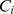 is randomly paired with the time series of trade volumes of a different company 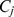. For each pair 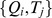 included in each randomly generated dataset, we compute the cross-correlation between 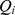 and 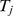.

We then compare the (macro-)average cross-correlation that we get for the real data with the average values obtained for the 1000 randomized datasets in which the queries of a company are always paired with the trades of another company. While the average result that we get for the original data is 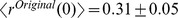, the values obtained for the test statistic when the random permutations are applied are much smaller. We find 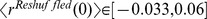. Therefore we get an empirical p-value of 0.001, meaning that the correlations observed on the real data are statistically significant at 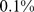.

We also check the significance of the correlations obtained for individual companies separately. Our goal here is to understand on a deeper level what companies are actually correlated with the corresponding queries, and which ones are not. We consider the two scenarios below.

1. In the first case, the null hypothesis is the following: *The correlation between trading volume of company 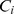 and query volume of the same company is not higher than the correlation between trading volume of company 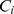 and query volume of some other company 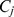.* For every company 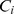, we compare the real data 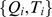 with the 1000 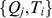 pairs where each 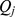 comes from one of the 1000 random datasets generated before. The test statistic that we use for the comparison is the same as before, that is, the cross-correlation coefficient 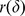 between the two time series forming any given pair. For every company 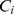, we compute the empirical p-value by taking the rank of the real test statistic 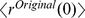 within the sorted order of the values computed from reshuffled data.
2. Similarly, in the second scenario, our null hypothesis is: *The correlation between query volume of company 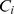 and trading volume of the same company is not higher than the correlation between query volume of company 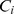 and trading volume of some other company 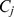.* Now, for any query-volume 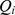, the real data is still given by the pair 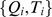. We compare this with the 1000 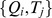 pairs where each 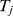 comes from a different random dataset. We calculate the cross-correlation between the two time-series included in every pair, and determine the p-values in the same way as above.

In both the scenarios taken into consideration, for most of the companies the test rejected 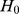. More specifically,

1. We got the minimum p-value 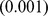 for 50 companies (out of 87). The p-value was 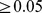 in 19 cases.
2. We got the minimum p-value 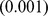 in 48 cases. The p-value was 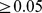 in 26 cases.

To summarize, we observe that for 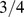 of the stocks the correlation between query volume and trading volume can not be explained by a simple global correlation between finance related search traffic and market activity in general.

It is worth noting that large p-values are related to companies for which poor correlation is present between query-log data and trading, maybe because of the large noise in the dataset.

**Table 10:** Age distribution of users.

Age Range	Fraction of Users
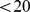	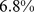
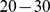	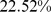
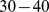	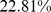
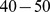	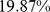
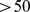	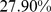

Average age distribution for a random sample collecting half of the data.

**Table 11:** Age distribution for NASDAQ-100 sample.

Age Range	Fraction of Users
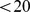	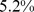
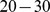	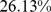
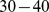	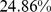
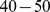	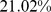
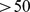	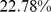

We observe some minor differences between the age of common users and the one of the users corresponding to queries belonging to NASDAQ-100 sample.

#### Correlation between query volume and volatility

Trading volume and volatility are correlated and volatility is autocorrelated. Therefore a source of the correlation between present query volume and future trading volume can be the autocorrelation component of volatility. Here we show that the origin of these correlations cannot be traced back to volatility. In order to perform such a task we compare the correlation between query volume and absolute price returns (i.e a proxy for the volatility) with the one between query volume and trading volume.

We define the *price return* of a day 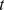 as follows:
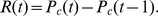
where 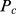 is the closing price of the day 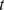. For each stock in our NASDAQ-100 *clean* list we compute the price returns and build three time series:

- The time series 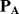 of the unsigned price returns: 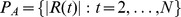

- The time series 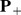 of the positive price returns: 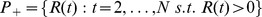

- The time series 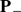 of the negative price returns: 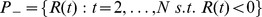

The time series 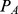 of the unsigned price returns has 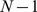 elements, being 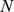 the length (number of days) of the time interval covered by our data (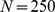).

Similarly to the experiments involving trading volumes, we compute for every stock the cross-correlation 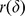 between the price returns and the query volume of the same company.

Fig. 5 (broken line) reports the cross-correlation function between the unsigned price returns and query volume. The average value of the basic cross-correlation at lag 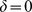 between query volume and price returns is 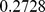. This result reflects the fact that in days when the prices of the NASDAQ-100 stocks exhibit a large variation (either positive or negative), there is a considerable amount of web search activity concerning the same stocks.

However, as shown in Fig. 5 the cross-correlation between query volume and volatility (broken line) is significantly smaller than the one between query volume and trading volume (solid line). Moreover the 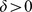 branch in case of volatility is equal or even smaller than the value observed in the 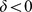 one. If the origin of the effect were due to the autocorrelation component of volatility, we would expect a similar behavior for both cross-correlation functions. These facts support the non-autocorrelated origin of the correlation between between todays query volume and future trading volume.

For the time series 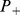 (positive returns) and 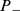 (negative returns), we only computed the cross-correlation between query volumes for lag 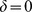. The reason is due to the fact that the time gap between two consecutive elements of those series is variable. The average correlations obtained for the 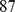 clean NASDAQ tickers are report in Table 8. The results are similar to ones we get for the unsigned price returns.

#### Granger Causality

The Granger-Causality test is widely used in time-series analysis to determine whether a time series 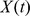 is useful in forecasting another time series 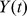. The idea is that if 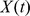 Granger-causes 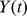 if 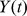 can be better predicted using both the histories of 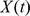 and 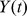 rather than using only the history of 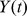. The test can be assessed by regressing 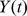 on its own time-lagged values and on those of 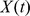. An F-test is then used to examine if the null hypothesis that 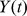 is not Granger-caused by 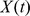 can be rejected.

In this work, we apply the Granger-causality test to analyze the relation between query volumes and trading volumes, and also between user volumes and trading volumes. Our aim is to prove that search activity related to a company, Granger-cause the trading volume on the company stock. However, we also want to verify whether the notion of Granger causality holds in the opposite direction. Hence, we apply the test in the two possible directions.

Again, we first consider all companies included in the NASDAQ-100 data set. However, given that we know from the previous analysis that in some cases the query volumes are very noisy and not related to the traded company they have been extracted for, we also perform the test on the smaller test of 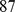 companies obtained through manual filtering.

Table 9 presents the results of the Granger-causality test. Each row in the table summarizes the outcome of an experiment. The table specifies the two available query-log time series (query volumes Q or user volumes U) compared with trading volume T (comparisons are always made for each company independently), the lag applied (expressed in terms of number of days), the direction in which the test is applied : 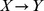 means that the null hypothesis 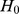 is “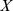 does not Granger-cause 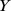
”. The last three columns provide a summary of the results obtained for all companies that are taken into consideration during the test. The fourth and fifth column respectively report the percentage of companies for which the null hypothesis was rejected with 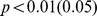. The last column reports the average reduction in RSS.

In all the cases, it can be observed that the 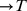 direction of the test is much stronger than the opposite direction 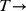. That is, we obtained stronger support for the case that time-series extracted from the query-log Granger-cause the trading volume of the same company, as opposed to trading volume Granger-causing query or user volumes. Especially this is the case when significance at 1% is required.

For instance, let us consider rows 9 and 11 in the Table 9. When the *clean* set of 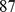 tickers is examined, we observe that in 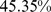 of the cases the null hypothesis (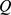 does not Granger-cause 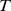) is rejected with 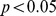, and for 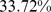 of the companies the same held with with 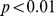. A much weaker result is obtained when the opposite direction is considered. Only for 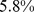 of the companies the null hypothesis could be rejected with 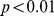.

As we have already observed in the cross-correlation experiment, we get slightly weaker results when considering user volumes. In fact observing line 11 of the table 9 we find that in 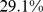 of the cases the trading volume 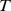 is Granger-caused by the user volume 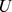 with probability greater than 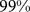. The average reduction in RSS is 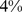.

In short, adding information about todays query volume reduces the average prediction error (in an autoregressive model) for tomorrows trading volume by about 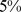. For half of the companies the reduction is statistically significant at 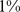, that is, both query volume and user volume Granger-causes the trading volume. We can also interpret this as follows: query/user volume helps to predict the trading volume, but the reverse does not hold.

It can be now argued that the Granger test, in principle, should be used only on series for which the error term in the regressions is gaussian. In this framework instead we are dealing with fat-tailed distribution underlying the query volume and trade volume series (see Supporting Information S1). However, in the next section we present a series of analyses which confirm the significance of the results found here. In particular, they all support the evidence that todays web search traffic is more informative on tomorrows trading activity than the reverse case.

#### Beyond Granger Causality

To study the anticipation effect and the power of search engine data for predicting stock trading volumes, we performed several statistical tests checking various hypotheses. The tests are detailed below.

### Test 1

To test if query volume can predict future trading volume, denoted 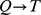, we use four different regression models:

1. 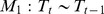:

We predict trading volume of tomorrow using trading volume of today.

2. 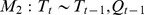:

We predict trading volume of tomorrow using both trading and query volume of today.

3. 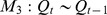:

We predict query volume of tomorrow using query volume of today.

4. 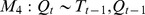:

We predict query volume of tomorrow using both trading and query volume of today.

Let 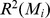 denote the sum of squared residuals for model 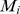. We define
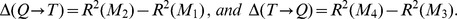



In other words 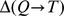 is the variation of 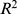 when we use 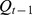 to predict 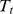 *in addition* to 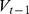. Likewise, 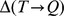 is the variation in 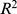 when 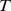 is added to an auto-regressive model of 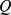.

Our aim is to test the following hypotheses:

1. Null-hypothesis 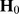: 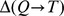 and 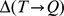 *are not significantly different*.
2. Alternative hypothesis 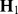 : 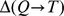 *is significantly larger than* 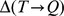.
3. Alternative hypothesis 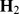: 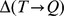 *is significantly larger than* 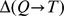.

To compare 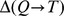 and 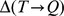, we apply a bootstrap procedure to estimate their distribution. We generate 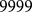 samples for 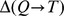 and 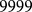 samples for 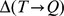, using the *case resampling* strategy. We denote by 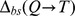 the bootstrap distribution of 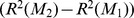, and by 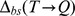 the bootstrap distribution of 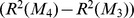.

Given 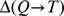 and 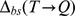, we can derive an empirical p-value of 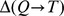 being larger than 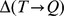. This p-value, which we denote by 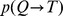, is computed as the the rank of 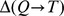 in the list of sorted 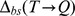 values divided by 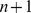, where 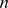 is the number of bootstrap samples. Depending on the chosen significance level, by the empirical p-value we can now reject 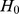, and support 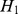.

We run this test for the list of *clean* NASDAQ-100 tickers. For 26 companies we obtain an empirical p-value lower than 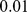: this result suggests that, for these companies, we can reject the null hypothesis at the significance level of 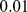, finding support for 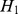.

In Supporting Information S2 (Test 1) we report the list of these companies, together with the respective p-values 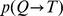 and 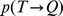. The third column of the table contains the value of the basic cross-correlation at lag 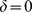 between query volume and trading volume.

We also test the opposite direction. To verify if there is any support for 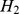, we took 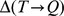 and 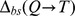, and use the same procedure as above to compute the empirical p-value of 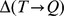 being larger than 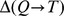. This time, all p-values 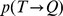 that we obtain for the 87 clean tickers are very large. In almost every case 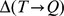 is smaller than the values in 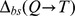. This suggests that trading volumes of today do not help in *predicting* query volumes of tomorrow.

In Supplementary S2 (Test 1) we report the ten tickers with the smallest 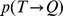 and we observe that even the smallest values are much larger than 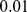, thus we not find any convincing support for 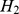.

### Test 2

The previous test is based on the idea of comparing the improvement in 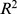 after adding information from the second time series to an auto-regressive model. The test that we present below is based on the direct comparison of the 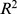 values of 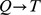 and 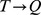.

We consider the two following regressive models:

1. 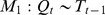


2. 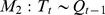


We perform the two regressions above, and compute the respective 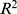 values, which we call 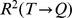 and 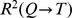. If 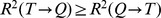, then we conclude 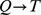, and viceversa.

To assess the significance of the test, we generate 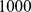 bootstrap vectors starting from the real data and applying random sampling with replacements. We compute 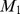 and 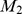 on the bootstrap vectors, obtain the corresponding residuals, and extract the 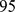-th percentiles 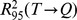 and 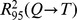, that is, the values such that, for 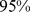 of the boostrap vectors, the sum of squared residual is below this values. Then we compare 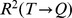 with 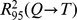, and 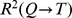 with 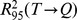.

We run this test on the clean set of NASDAQ-100 tickers. For a significance level of 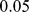, the outcome is the following:

- 61 companies with a *significant* difference at 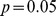 between 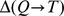 and 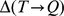 values: 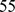 support 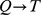, and 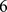 support 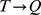 (These are: joyg, lltc, rost, teva, vrsn, vrtx).
- 26 companies have no *significant* difference between the two directions (see Supporting Information S2 (Test 2)).

### Test 3

In this test we again consider the four regression models that are used for the first test:

1. 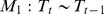:

We predict trading volume of tomorrow using the trading volume of today.

2. 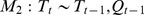:

We predict trading volume of tomorrow using both trading and query volume of today.

3. 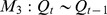:

We predict query volume of tomorrow using the query volume of today.

4. 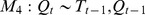:

We predict query volume of tomorrow using both trading and query volume of today.

We consider the following hypothesis:

1. Null-hypothesis 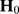: 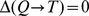

2. Alternative hypothesis 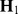 : 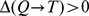.

To test if 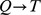, we compute the regression models 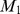 and 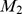, and derive the corresponding residuals 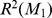 and 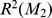. We then compute 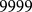 bootstrap estimates of 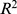 both for 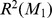 and 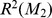. Next we compare these two bootstrap samples by applying the Mann-Whitney U test, also known as the Wilcoxon rank-sum test.

The test is aimed at assessing whether one of two samples of independent observations tends to have larger values than the other. It is based on the null-hypothesis of the two samples having equal medians.

We also test the opposite direction 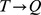. We compute the regression models 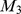 and 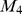, and the corresponding residuals 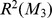 and 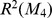. We compute 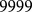 bootstrap estimates of 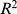 both for 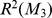 and 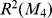, and we apply again the Mann-Whitney U test. For the 87 clean NASDAQ-100 tickers, we get the following results (see Supporting Information S2(Test 3)):

- Only 3 out of 87 clean Nasdaq tickers are not significant at 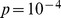 when testing for 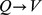. These are LINTA 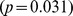, CHKP 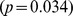 and FISV 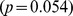.
- In the other direction, 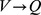, only 19 tickers are not significant at 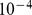.
- In every other case the p-value is approximately 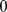. This might be due to the Mann-Whitney test being better suited for small sample sizes.

### Analysis of Users’ Behavior

We now investigate the typical behavior of search-engine users who issue queries related to NASDAQ-100 tickers. In particular, our goal was to answer to the following questions:

- What does a typical user search for?
- Does a user look for many different tickers, or just for a few ones or even one?
- Does a user ask the same question repeatedly on a certain regular basis, or sporadically?
- Can we identify groups of users with a similar behavior?

First, we compute the distribution of the number of distinct tickers that any user looks at within a month. We then obtain an average monthly distribution by averaging over the 12 months in our period of observation, as shown in Fig. 6. We also compute the distribution of the number of distinct tickers that any user looked at within the whole year, as shown in Fig. 6. The distributions show very clearly that the overwhelming majority of the users search only for one ticker, not only within one month, but also within the whole year.

To further characterize the behavior of users with respect to this one ticker they look for, we then check how frequently people look for their favorite ticker, and if they search it regularly over time (once a day, once a week, once a month). To conduct this study we focus on three of the tickers characterized by the highest cross-correlation between query volumes and trading volumes: AAPL (Apple Inc.), AMZN (Amazon.com), and NFLX (NetFlix, Inc.).

For each of these tickers, we consider the set of users who made at least one search related to the ticker during the whole year, and we compute the distribution of the number of days on which any users searched the ticker. We first consider, separately, the distribution for each month, and then we take the average over the twelve months. We also compute the distribution over the whole year. The yearly and monthly distributions for the three tickers are shown in Figs. 7, 8, 9. Surprisingly, in all the cases considered, a major fraction of the users (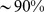) looks at their favorite ticker only one time during a month and the whole year.

Given the correlation and the anticipation of query volumes over trading volumes described in the previous section one could expect to observe a significant fraction of users regularly querying for a stock and doing so more frequently in coincidence of peaks of trading activity. In contrast, the typical behavior of users suggests the profile of people who are not financial experts nor regularly following the market trend. It is thus remarkable that, despite emerging from the uncoordinated action of “normal” people, the query activity still works well as a proxy to anticipate market trends.

Finally, for the subset of users who have a registered Yahoo! profile, we also analyze the personal data that they provide concerning gender, age, country. To check if the users who seek NASDAQ-100 tickers behave differently from the rest of the Yahoo! users, we compare the set of registered users who submitted at least one query related to a NASDAQ-100 ticker with a random sample containing half of the registered users who were tracked in the log during the whole year. We compute the distributions of the demographic properties for the two aforementioned set of users.

Table 10 and Table 11 respectively report the age distribution for the random sample and for the set of NASDAQ-100 users. It is worth to observe that the population of NASDAQ-100 users contains a smaller fraction of old people. Altogether, 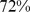 of the NASDAQ-100 users are people in working age, while this fraction is equal to 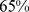 in the other sample, which we assume to be a fair representative of the whole set of Yahoo! users.

For what concerns gender, we observe that 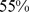 of the NASDAQ-100 users are males, and 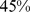 are females. The random sample has 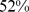 of male users, and 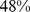 of females. Thus the set of users who searched NASDAQ-100 tickers includes a slightly larger fraction of males.

For the country distribution, we get similar finding on the two set of users. In both cases, the top-5 states which the users come from are California (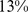), Texas (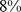), New York (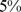), Florida (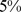) and Illinois (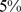). These fractions are expected, given that the aforementioned states are the most populated within the United States.

## Supporting Information

Supporting Information S1 **Detailed analysis and results of all the NASDAQ-100 stocks and of the 87 clean stocks whose average cross correlation functions are presented in the main text.**
(PDF)

Supporting Information S2 **Detailed results of the three tests proposed, beyond Granger test, to validate the finding that query volumes anticipate trading volumes.**
(PDF)

## References

[1] Mitchell T (2009). Mining our reality.. Science.

[2] Vespignani A (2009). Predicting the behavior of techno-social systems.. Science.

[3] Evans J, Rzhetsky A (2010). Machine science.. Science.

[4] Lazer D, Pentland A, Adamic L, Aral S, Barabasi A L (2009). Life in the network: the coming age of computational social science.. Science.

[5] Gonzalez M, Hidalgo C, Barabasi A L (2008). Understanding individual human mobility patterns.. Nature.

[6] Choi H, Varian H (2009). Predicting the present with google trends.. Technical report.

[7] Goel S, Hofman J, Lahaie S, Pennock D, Watts D (2010). Predicting consumer behaviour with web search. Proc. Natl. Acad. Sci.. USA.

[8] Golder S, Macy M (2011). Diurnal and seasonal mood vary with work, sleep, and daylength across diverse cultures.. Science.

[9] Preis T, Moat H S, Stanley H E, Bishop S R (2012). Quantifying the Advantage of Looking Forward.. Nature Scientific Report.

[10] Ginzberg J, Mohebi M, Patel R, Brammer L, Smolinski M (2009). Detecting influenza epi-demics using search engine query data.. Nature.

[11] Saavedra S, Hagerty K, Uzzi B (2011). Synchronicity, instant messaging, and performance among financial traders. Proc. Natl. Acad. Sci.. USA.

[12] Preis T, Reith D, Stanley H E (2010). Complex dynamics of our economic life on different scales: insights from search engine query data. Phil. Trans. R. Soc.. A.

[13] Bollen J, Mao H, Zeng X J (2011). Twitter mood predicts the stock market.. J Comput Sci.

[14] Bouchaud J-P (2009). The (unfortunate) complexity of the economy.. Physics World.

[15] Haldane A G, May R M (2011). Systemic risk in banking ecosystems.. Nature.

[16] Schweitzer F, Fagiolo G, Sornette D, Vega-Redondo F, Vespignani A (2009). Economic Networks: The New Challenges.. Science.

[17] Bouchaud J-P (2008). Economics needs a scientific revolution.. Nature.

[18] Asur S, Huberman B A (2010). Predicting the Future With Social Media.. arXiv.

[19] Podobnik B, Horvatic D, Petersen A, Stanley H E (2009). Cross-correlations between volume change and price change. Proc. Natl. Acad. Sci.. USA.

[20] Plerou V, Gopikrishnan P, Rosenow B, Amaral L, Stanley H E (2000). Econophysics: financial time series from a statistical physics point of view.. Physica A.

[21] Yamasaki K, Muchnik L, Havlin S, Bunde A, Stanley H E (2005). Scaling and memory in volatility return intervals in financial markets.. Proc Natl Acad Sci USA.

[22] Onnela J P, Chakraborti A. Kaski K, Kertesz J, Kanto A (2003). Asset trees and asset graphs in financial markets.. Physica Scripta.

[23] Onnela J P, Chakraborti A, Kaski K, Kertesz J (2002). Dynamic asset trees and portfolio analysis.. Eur Phys J B.

[24] Garlaschelli D, Battiston S, Castri M, Servedio V D P, Caldarelli G (2005). The scale-free topology of market investments.. Physica A.

[25] Cont R (2001). Empirical properties of asset returns: stylized facts and statistical issues.. Quantitative Finance.

[26] Liu Y, Gopikrishnan P, Cizeau P, Meyer M, Peng C-K (1999). Statistical properties of the volatility of price fluctuations. Phys. Rev.. E.

[27] Bouchaud J P, Potters M (2009). Theory of Financial Risk and Derivative Pricing: From Statistical Physics to Risk Management.. Cambridge University Press, 2nd edition.

[28] Granger C (1969). Investigating causal relations by econometric models and cross-spectral methods.. Econometrica.

[29] Easley D, Kleinberg J (2010). Networks, Crowds, and Markets: Reasoning About a Highly Connected World.. Cambridge University Press.

[30] Balcan D, Colizza V, Goncalves B, Hu H, Ramasco J (2009). Multiscale mobility networks and the spatial spreading of infectious diseases. Proc. Natl. Acad. Sci.. USA.

[31] Pastor-Satorras R, Vespignani A (2010). Patterns of complexity.. Nature Physics.

[32] Colizza V, Pastor-Satorras R, Vespignani A (2007). Reaction-diffusion processes and metapopulation models in heterogeneous networks.. Nature Physics.

