# Supplementary material for: Web Search Queries Can Predict Stock Market Volumes
Source: PLoS One. 2012 Jul 19;7(7):e40014. doi: 10.1371/journal.pone.0040014 (PMC3400625; doi:10.1371/journal.pone.0040014)
Supplement: Supporting Information S1 — Detailed analysis and results of all the NASDAQ-100 stocks and of the 87 clean stocks whose average cross correlation functions are presented in the main text. (PDF) [file pone.0040014.s001.pdf]

SUPPORTING INFORMATION S1: Web search queries can predict stock market volumes.

Ilaria Bordino[1], Stefano Battiston[2], Guido Caldarelli[3,4,5], Matthieu Cristelli[3,*], Antti Ukkonen[1], Ingmar Weber[1]
1 Yahoo! Research, Barcelona, Spain
2 ETH Chair of System Design, Zurich, Switzerland
3 Inst. of Complex Systems CNR, "Sapienza" University, Rome, Italy
4 London Institute for Mathematical Sciences, London, United Kingdom
5 IMT - Institute for Advanced Studies, Lucca, Italy
∗ E-mail: matthieu.cristelli@roma1.infn.it

1 Data Analysis and Results: all the NASDAQ-100 stocks

In this section we report the complete results of the stocks on which the averages shown and discussed in the main paper are performed.

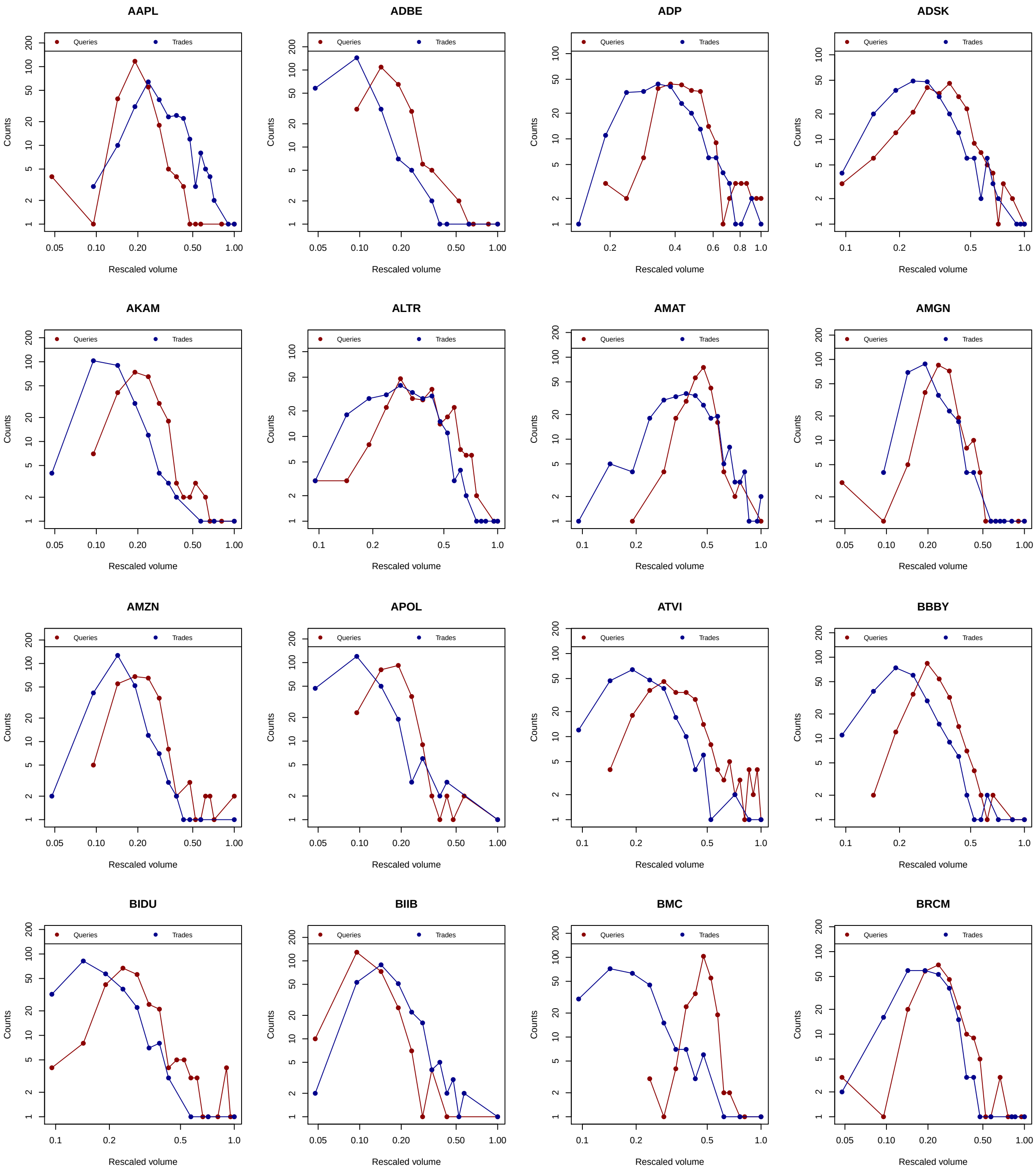

Figure S 1. Histograms of trading volumes and query volumes for all the 87 clean stocks. Most of the distributions appear to be fat-tailed. All the series have been rescaled dividing them by their maximum value.

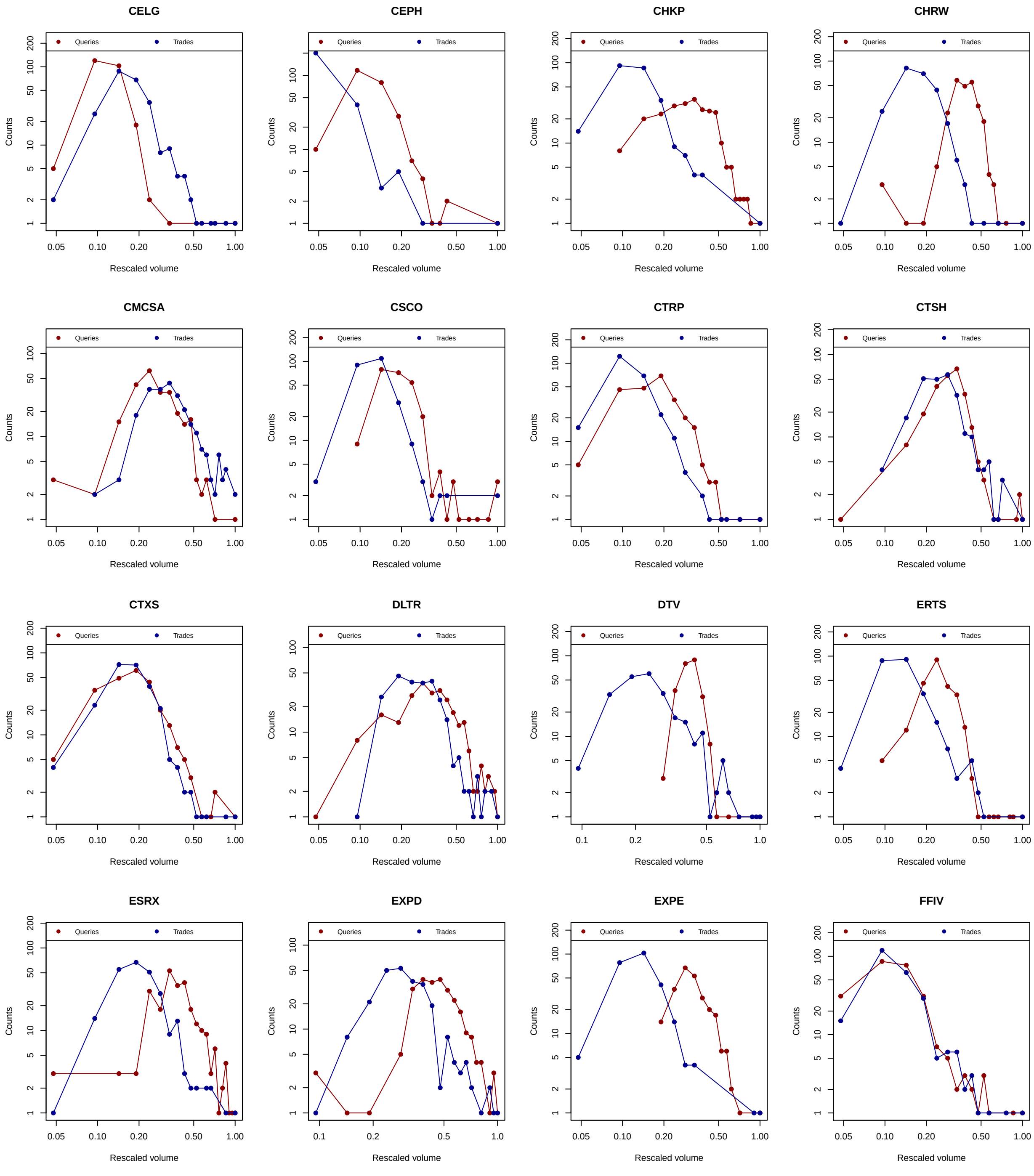

Figure S 2. Histograms of trading volumes and query volumes for all the 87 clean stocks. Most of the distributions appear to be fat-tailed. All the series have been rescaled dividing them by their maximum value.

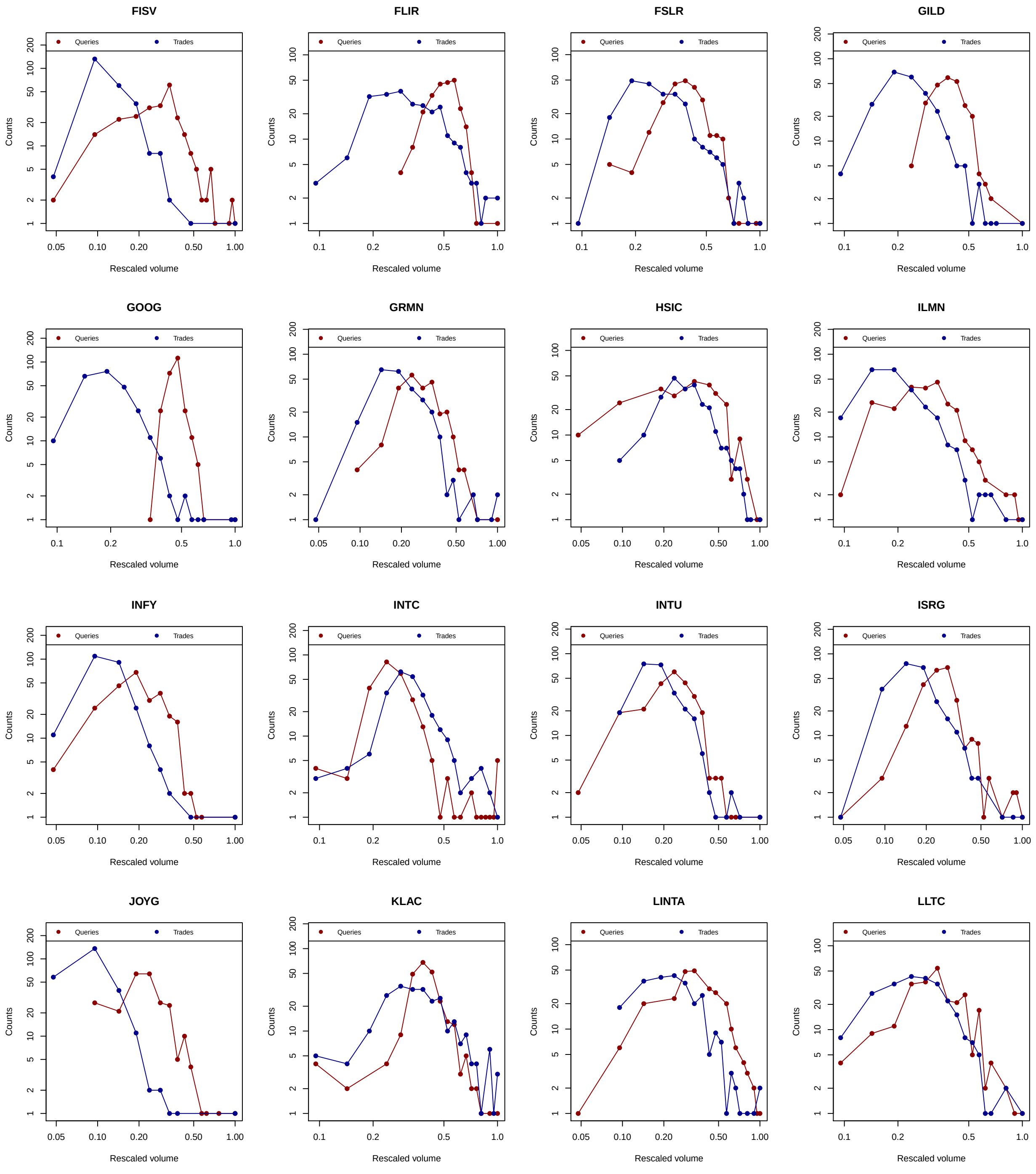

Figure S 3. Histograms of trading volumes and query volumes for all the 87 clean stocks. Most of the distributions appear to be fat-tailed. All the series have been rescaled dividing them by their maximum value.

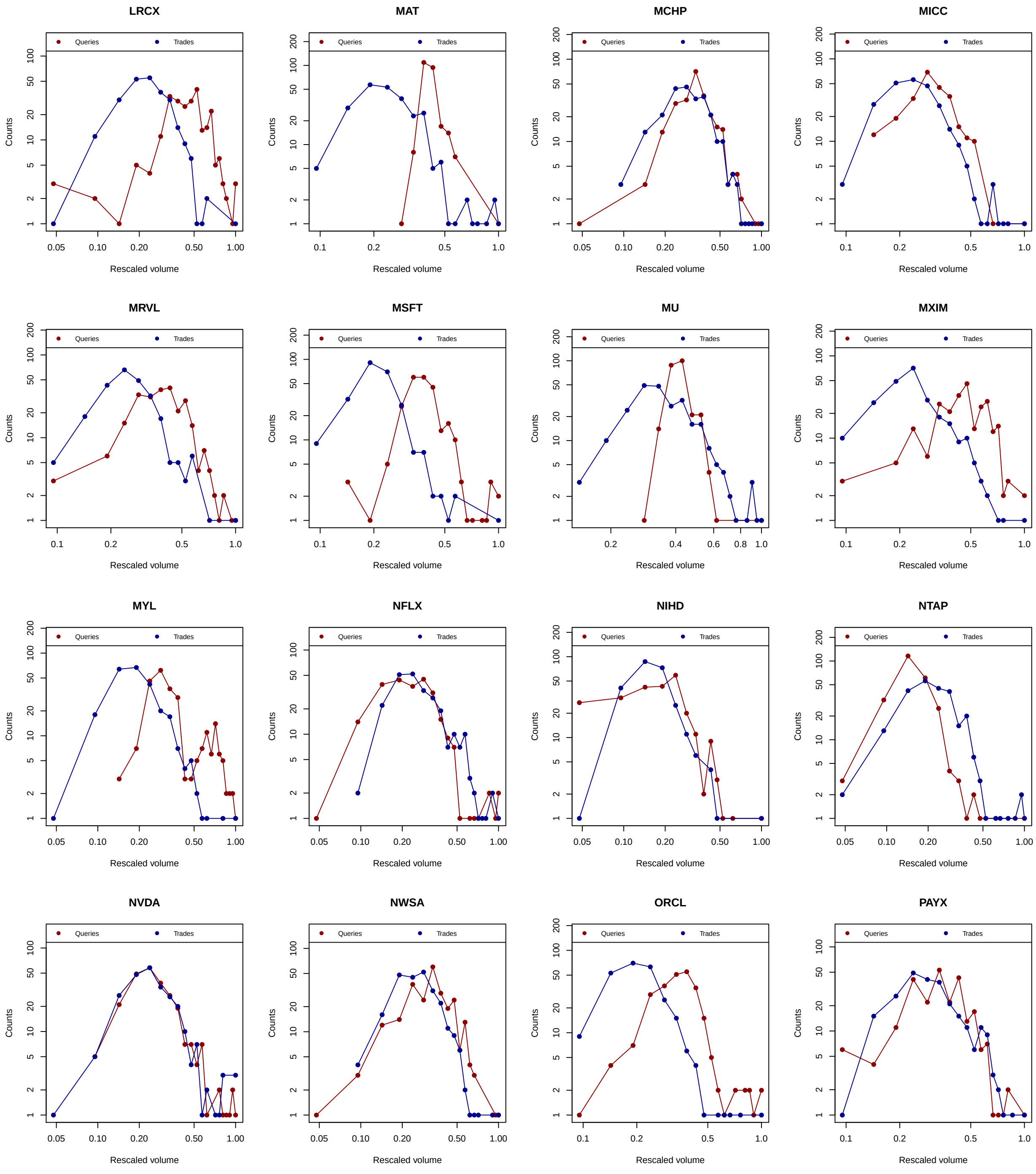

Figure S 4. Histograms of trading volumes and query volumes for all the 87 clean stocks. Most of the distributions appear to be fat-tailed. All the series have been rescaled dividing them by their maximum value.

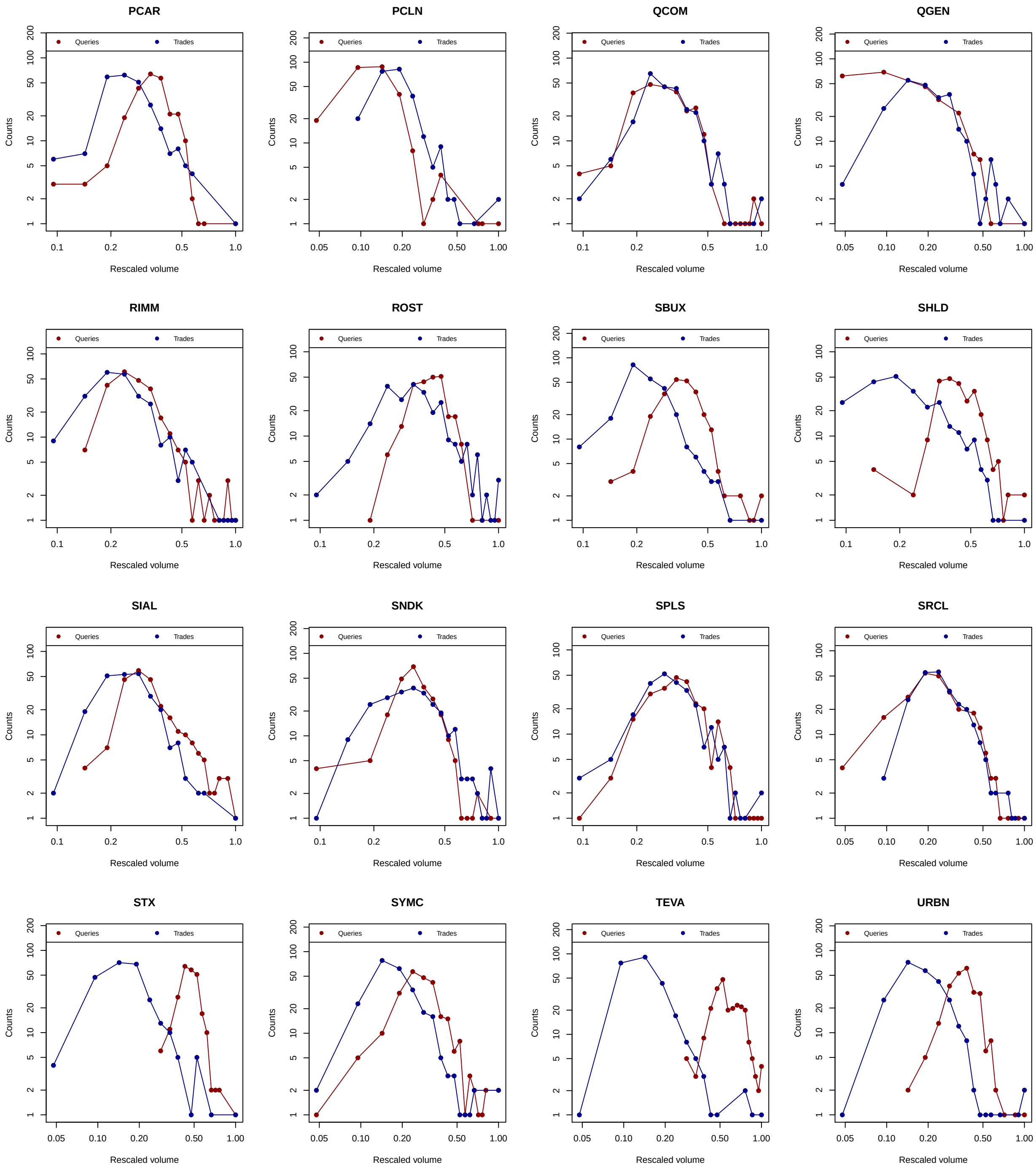

Figure S 5. Histograms of trading volumes and query volumes for all the 87 clean stocks. Most of the distributions appear to be fat-tailed. All the series have been rescaled dividing them by their maximum value.

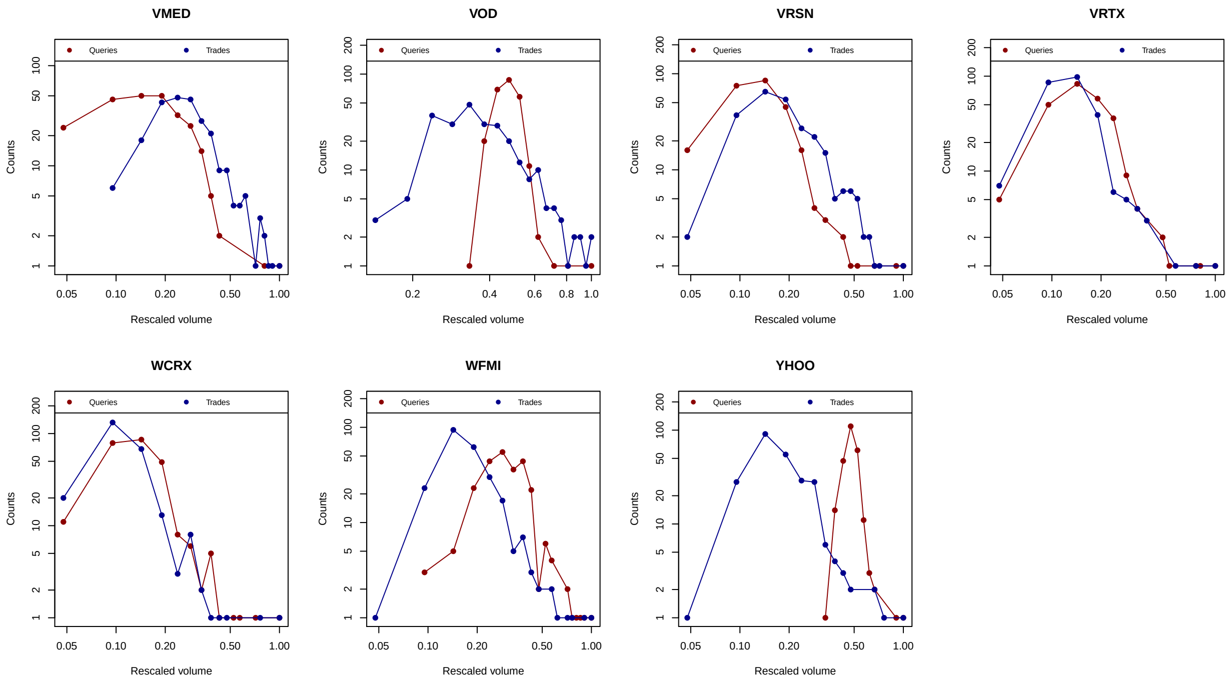

Figure S 6. Histograms of trading volumes and query volumes for all the 87 clean stocks. Most of the distributions appear to be fat-tailed. All the series have been rescaled dividing them by their maximum value.

Table S 1. Values of cross-correlation functions for the 87 *clean* stocks.

**Ticker**	δ =**-5**	δ =**-4**	δ =**-3**	δ =**-2**	δ =**-1**	δ =**0**	δ =**1**	δ =**2**	δ =**3**	δ =**4**	δ =**5**
AAPL	0.03	0.07	0.07	0.13	0.30	0.58	0.40	0.19	0.13	0.05	0.04
ADBE	0.08	0.12	0.14	0.19	0.47	0.83	0.51	0.19	0.09	0.10	0.11
ADP	-0.19	-0.21	-0.23	-0.16	-0.15	-0.15	-0.18	-0.19	-0.15	-0.14	-0.15
ADSK	-0.16	-0.15	-0.03	0.01	0.09	0.19	0.26	0.03	-0.09	-0.04	-0.09
AKAM	-0.04	-0.06	0.03	0.07	0.22	0.72	0.49	0.20	0.11	0.02	-0.01
ALTR	0.34	0.40	0.40	0.37	0.42	0.55	0.53	0.39	0.41	0.37	0.38
AMAT	0.05	0.04	0.03	0.03	0.05	0.10	0.15	0.04	-0.01	-0.07	-0.10
AMGN	-0.02	0.03	0.03	0.10	0.19	0.36	0.35	0.18	0.14	0.11	-0.02
AMZN	-0.07	-0.03	-0.04	-0.02	0.13	0.48	0.43	0.04	-0.02	0.01	-0.02
APOL	0.02	0.06	0.10	0.21	0.43	0.79	0.55	0.22	0.12	0.07	0.03
ATVI	-0.04	0.05	0.10	0.16	0.27	0.39	0.39	0.22	0.23	0.18	0.10
BBBY	0.04	0.19	0.13	0.12	0.21	0.43	0.39	0.14	0.09	0.07	0.14
BIDU	0.10	0.09	0.12	0.19	0.32	0.49	0.42	0.16	0.11	0.05	0.04
BIIB	0.06	0.09	0.13	0.10	0.21	0.59	0.23	0.20	0.10	0.07	0.09
BMC	0.05	0.14	0.08	0.19	0.04	0.17	0.20	0.21	0.18	0.10	0.12
BRCM	-0.02	-0.02	0.04	0.09	0.22	0.53	0.45	0.15	0.07	0.05	0.01
CELG	0.00	0.03	0.01	0.02	0.20	0.65	0.29	0.03	0.05	0.04	0.05
CEPH	0.16	0.26	0.22	0.14	0.32	0.80	0.44	0.24	0.12	0.13	0.15
CHKP	-0.03	-0.06	-0.04	-0.07	0.06	0.09	0.03	-0.02	-0.05	-0.03	-0.01
CHRW	0.03	0.12	0.07	-0.05	0.00	0.16	0.23	0.07	0.05	-0.06	-0.06
CMCSA	-0.20	-0.16	-0.15	-0.16	-0.10	0.02	-0.05	-0.12	-0.12	-0.13	-0.11
CSCO	0.04	0.07	0.13	0.36	0.53	0.74	0.63	0.34	0.26	0.17	0.12
CTRP	-0.02	0.08	0.01	0.11	0.19	0.57	0.26	0.06	0.03	0.04	0.06
CTSH	-0.06	0.02	0.07	0.11	0.15	0.38	0.12	0.07	0.06	0.01	-0.05
CTXS	0.11	0.15	0.14	0.18	0.26	0.55	0.35	0.14	0.14	0.10	0.06
DLTR	0.07	0.16	0.17	0.14	0.23	0.42	0.25	0.24	0.15	0.07	0.04
DTV	0.04	0.02	0.06	0.09	0.05	0.03	0.03	-0.05	0.05	0.03	-0.01
ERTS	0.06	0.17	0.22	0.24	0.34	0.62	0.53	0.18	0.05	-0.02	0.02
ESRX	0.14	0.23	0.17	0.21	0.21	0.43	0.31	0.17	0.16	0.09	0.05
EXPD	0.01	0.07	0.08	0.09	0.24	0.37	0.31	0.22	0.18	0.16	0.11
EXPE	0.10	0.14	0.13	0.16	0.27	0.52	0.40	0.17	0.17	0.19	0.15
FFIV	0.06	0.06	0.13	0.21	0.35	0.65	0.56	0.33	0.21	0.14	0.13
FISV	0.03	0.01	-0.05	0.08	0.08	0.28	0.12	0.02	0.02	0.06	0.11
FLIR	-0.13	-0.11	-0.10	-0.15	-0.14	-0.13	-0.09	-0.12	-0.16	-0.16	-0.16
FSLR	0.01	0.05	0.02	0.13	0.29	0.55	0.44	0.18	0.12	0.02	-0.01
GILD	0.03	0.13	0.13	0.12	0.13	0.18	0.14	0.06	0.08	0.03	0.02
GOOG	-0.09	0.03	-0.00	0.01	0.04	0.02	0.09	-0.02	-0.05	-0.01	-0.07
GRMN	0.07	0.10	0.07	0.05	0.23	0.46	0.24	0.12	0.09	0.07	0.08
HSIC	-0.20	-0.18	-0.13	-0.10	0.04	0.07	-0.02	-0.07	-0.00	-0.03	-0.16
ILMN	-0.01	0.06	0.12	0.16	0.20	0.40	0.39	0.31	0.27	0.21	0.14
INFY	-0.05	-0.09	0.02	0.06	0.14	0.53	0.20	0.06	0.10	0.03	0.00
INTC	0.07	0.04	0.00	0.05	0.18	0.44	0.40	0.14	0.09	0.05	0.03
INTU	-0.08	-0.10	-0.10	-0.07	-0.00	0.31	0.22	0.10	0.03	-0.05	-0.10
ISRG	0.07	0.13	0.18	0.21	0.38	0.67	0.64	0.29	0.20	0.11	0.05

Table S 2. Values of cross-correlation functions for the 87 *clean* stocks.

**Ticker**	δ =**-5**	δ =**-4**	δ =**-3**	δ =**-2**	δ =**-1**	δ =**0**	δ =**1**	δ =**2**	δ =**3**	δ =**4**	δ =**5**
JOYG	0.00	0.05	0.13	0.10	0.17	0.27	0.13	0.09	0.06	0.09	0.05
KLAC	0.36	0.40	0.40	0.46	0.45	0.43	0.49	0.46	0.47	0.43	0.39
LINTA	0.06	-0.04	0.02	0.00	0.04	0.04	0.01	0.01	0.02	0.04	-0.06
LLTC	0.16	0.22	0.18	0.22	0.32	0.39	0.32	0.21	0.13	0.10	0.12
LRCX	-0.01	0.00	-0.02	0.04	0.17	0.24	0.20	0.16	0.14	-0.03	0.00
MAT	0.06	0.21	0.07	0.10	0.09	0.04	0.06	0.03	-0.02	0.05	0.02
MCHP	0.22	0.21	0.22	0.23	0.23	0.24	0.32	0.25	0.18	0.14	0.10
MICC	0.04	0.10	0.06	0.14	0.16	0.21	0.17	0.06	0.04	0.02	0.03
MRVL	-0.06	0.09	0.02	0.06	0.12	0.40	0.37	0.02	0.01	0.03	-0.00
MSFT	-0.09	-0.02	-0.06	0.02	0.17	0.42	0.35	0.02	0.05	0.04	0.09
MU	-0.13	-0.03	-0.05	-0.07	-0.06	-0.05	-0.05	-0.10	-0.08	-0.07	-0.15
MXIM	0.11	0.09	0.19	0.18	0.22	0.29	0.11	-0.04	0.03	0.03	0.01
MYL	-0.10	-0.07	-0.07	-0.10	-0.11	-0.07	-0.07	-0.06	-0.07	-0.04	-0.01
NFLX	0.10	0.16	0.16	0.24	0.47	0.68	0.54	0.25	0.19	0.16	0.13
NIHD	0.10	0.11	0.20	0.24	0.30	0.56	0.34	0.25	0.15	0.11	0.09
NTAP	-0.06	0.02	0.01	0.06	0.26	0.61	0.46	0.18	0.09	0.09	0.11
NVDA	0.23	0.36	0.38	0.46	0.56	0.79	0.68	0.47	0.42	0.38	0.29
NWSA	-0.04	0.03	-0.03	-0.10	-0.01	0.06	0.09	-0.04	-0.04	-0.03	-0.08
ORCL	0.09	0.17	0.09	0.07	0.23	0.52	0.43	0.13	0.16	0.10	0.03
PAYX	0.06	0.08	-0.00	0.05	0.04	0.04	0.03	0.00	-0.00	-0.06	-0.03
PCAR	0.04	0.14	0.14	0.15	0.16	0.27	0.28	0.14	0.14	0.15	0.06
PCLN	-0.10	-0.04	-0.03	0.01	0.20	0.51	0.37	0.06	-0.01	-0.06	-0.06
QCOM	-0.15	-0.11	-0.12	-0.06	0.09	0.24	0.15	-0.06	-0.10	-0.09	-0.14
QGEN	0.09	0.09	0.06	0.11	0.09	0.35	0.31	0.15	0.13	0.10	0.21
RIMM	0.03	0.12	0.11	0.14	0.31	0.66	0.58	0.24	0.20	0.11	0.05
ROST	-0.22	-0.12	-0.15	-0.11	-0.17	-0.08	-0.12	-0.10	-0.13	-0.20	-0.16
SBUX	-0.08	0.03	0.08	0.09	0.19	0.41	0.25	0.18	0.11	0.06	-0.04
SHLD	0.10	0.14	0.11	0.22	0.21	0.38	0.26	0.17	0.15	0.15	0.07
SIAL	-0.05	0.00	-0.02	-0.03	-0.05	-0.05	0.00	-0.01	0.01	-0.01	0.02
SNDK	0.04	0.02	0.11	0.23	0.30	0.45	0.37	0.09	0.13	0.11	0.01
SPLS	-0.19	-0.17	-0.17	-0.17	-0.04	0.11	0.02	-0.15	-0.16	-0.18	-0.11
SRCL	0.05	0.02	-0.04	0.01	0.12	0.27	0.24	0.21	0.08	0.05	0.05
STX	0.11	0.23	0.16	0.20	0.24	0.37	0.31	0.13	0.03	0.05	-0.01
SYMC	-0.00	0.02	0.11	0.17	0.25	0.58	0.44	0.21	0.14	0.04	0.04
TEVA	0.15	0.17	0.23	0.24	0.29	0.40	0.24	0.21	0.17	0.14	0.11
URBN	0.00	0.08	0.10	0.09	0.17	0.37	0.32	0.14	0.10	0.01	-0.01
VMED	-0.09	-0.14	-0.13	-0.13	-0.12	-0.09	-0.09	-0.13	-0.09	-0.08	-0.12
VOD	0.10	0.10	0.07	0.10	0.11	0.17	0.15	0.13	0.17	0.15	0.03
VRSN	0.00	0.05	0.01	0.18	0.44	0.56	0.40	0.26	0.22	0.18	0.16
VRTX	0.02	0.14	0.32	0.42	0.30	0.50	0.24	0.07	0.19	0.14	0.16
WCRX	0.05	0.06	0.06	0.07	0.17	0.51	0.23	0.11	0.05	0.05	0.01
WFMI	-0.00	-0.05	-0.01	0.06	0.23	0.45	0.31	0.03	-0.03	-0.06	-0.06
YHOO	0.06	0.15	0.15	0.16	0.23	0.38	0.25	-0.02	-0.00	0.02	-0.03

The values of the cross-correlation function $r(\delta)$ for $\delta > 0$ are on average larger than the value of $r(-\delta)$. In fact considering only the stocks for which $r(1) > 0$ (there are 8 stocks for which $r(1) < 0$) we observe that for 68 stocks it holds that $r(1) > r(-1)$ while for the remaining 11 stocks we observe $r(1) \leq r(-1)$.

Table S 3. Values of cross-correlation functions for the discarded stocks from the original set.

**Ticker**	δ =**-5**	δ =**-4**	δ =**-3**	δ =**-2**	δ =**-1**	δ =**0**	δ =**1**	δ =**2**	δ =**3**	δ =**4**	δ =**5**
CERN	-0.03	-0.02	0.03	0.02	0.00	0.03	-0.01	-0.00	0.03	0.02	-0.06
COST	-0.28	-0.21	-0.18	-0.19	-0.20	-0.17	-0.06	-0.07	-0.16	-0.10	-0.11
DELL	-0.05	-0.04	-0.01	0.01	-0.11	-0.11	-0.05	-0.04	-0.05	-0.02	-0.07
EBAY	-0.08	-0.07	-0.10	-0.16	-0.21	-0.18	-0.10	-0.18	-0.17	-0.20	-0.20
FAST	-0.14	-0.12	-0.12	-0.12	-0.06	-0.08	-0.07	-0.14	-0.11	-0.10	-0.13
FLEX	-0.12	0.05	-0.15	-0.19	-0.20	-0.09	-0.08	-0.16	-0.20	-0.18	-0.18
LIFE	-0.07	0.01	-0.04	-0.01	-0.09	-0.08	-0.05	-0.06	0.01	0.11	0.11
ORLY	0.01	-0.01	-0.04	-0.00	0.04	0.11	0.14	0.07	0.09	0.07	0.10
WYNN	-0.01	0.03	0.06	0.08	-0.02	0.02	0.10	-0.03	0.00	0.04	-0.08
XLNX	0.03	-0.00	0.02	0.06	0.09	0.14	0.14	-0.03	-0.03	-0.12	-0.03
XRAY	-0.12	-0.12	-0.22	-0.18	-0.18	-0.10	-0.08	-0.11	-0.05	-0.07	-0.12

Most of the query volumes associated to these tickers can be traced back to non-financial origin.

Table S 4. Cross-correlation coefficient $r(0)$ between query and trading volumes after removing largest events.

**Ticker**	$r(0)$	$r(0)-$Top5	$r(0)-$Top 10
AAPL	0.5826	0.4769	0.4481
ADBE	0.8326	0.5196	0.3137
ADP	-0.1456	-0.1246	-0.1120
ADSK	0.1933	0.1966	0.1795
AKAM	0.7243	0.4893	0.4059
ALTR	0.5546	0.5229	0.4956
AMAT	0.1014	0.1145	0.0732
AMGN	0.3563	0.3165	0.3138
AMZN	0.4838	0.3356	0.1784
APOL	0.7927	0.5547	0.4614
ATVI	0.3854	0.3291	0.2410
BBBY	0.4300	0.2963	0.2290
BIDU	0.4891	0.3355	0.3001
BIIB	0.5877	0.3449	0.3320
BMC	0.1676	0.1508	0.1600
BRCM	0.5342	0.2219	0.2338
CELG	0.6508	0.3171	0.1942
CEPH	0.7959	0.3208	0.2339
CHKP	0.0939	0.0838	0.0808
CHRW	0.1619	0.0559	0.0530
CMCSA	0.0242	-0.0299	-0.0456
CSCO	0.7352	0.5614	0.5014
CTRP	0.5659	0.3203	0.2963
CTSH	0.3791	0.2344	0.1756
CTXS	0.5522	0.3525	0.2897
DLTR	0.4243	0.3567	0.2830
DTV	0.0308	0.0860	0.1069
ERTS	0.6190	0.4764	0.3225
ESRX	0.4319	0.3371	0.2189
EXPD	0.3749	0.3186	0.3048
EXPE	0.5177	0.3473	0.2712
FFIV	0.6534	0.5410	0.5034
FISV	0.2754	0.0568	0.0589
FLIR	-0.1267	-0.1959	-0.1932
FSLR	0.5464	0.4577	0.4020
GILD	0.1775	0.1901	0.2013
GOOG	0.0199	-0.0440	-0.1211
GRMN	0.4564	0.2749	0.2763
HSIC	0.0706	-0.0198	0.0053
ILMN	0.4004	0.3020	0.3062
INFY	0.5338	0.1080	0.0469
INTC	0.4357	0.3178	0.3067
INTU	0.3096	0.0262	-0.0665
ISRG	0.6683	0.5432	0.5590

Table S 5. Cross-correlation coefficient $r(0)$ between query and trading volumes after removing largest events.

**Ticker**	$r(0)$	$r(0)-$Top5	$r(0)-$Top 10
JOYG	0.2660	0.2147	0.1841
KLAC	0.4307	0.4260	0.4305
LINTA	0.0446	-0.0066	0.0156
LLTC	0.3896	0.3286	0.2471
LRCX	0.2424	0.2749	0.2157
MAT	0.0441	-0.0104	-0.1008
MCHP	0.2411	0.1850	0.2042
MICC	0.2099	0.1548	0.1556
MRVL	0.3966	0.2554	0.2236
MSFT	0.4216	0.3808	0.3361
MU	-0.0458	-0.0440	-0.0411
MXIM	0.2948	0.2009	0.1671
MYL	-0.0665	-0.0871	-0.1243
NFLX	0.6757	0.6314	0.6253
NHID	0.5553	0.3644	0.2925
NTAP	0.6102	0.4173	0.2906
NVDA	0.7856	0.6866	0.6481
NWSA	0.0620	0.0729	0.0794
ORCL	0.5156	0.3493	0.3218
PAYX	0.0365	0.1071	0.1005
PCAR	0.2725	0.1737	0.1798
PCLN	0.5091	0.3054	0.2211
QCOM	0.2444	0.0681	0.0853
QGEN	0.3508	0.2262	0.2092
RIMM	0.6587	0.5946	0.5564
ROST	-0.0847	-0.1247	-0.1385
SBUX	0.4095	0.3263	0.2085
SHLD	0.3826	0.3706	0.3563
SIAL	-0.0475	-0.0053	-0.0396
SNDK	0.4510	0.3761	0.3404
SPLS	0.1144	-0.0184	-0.0031
SRCL	0.2695	0.1365	0.1023
STX	0.3738	0.2979	0.2242
SYMC	0.5761	0.3703	0.4122
TEVA	0.4005	0.2934	0.3379
URBN	0.3714	0.2841	0.2409
VMED	-0.0938	-0.1070	-0.0922
VOD	0.1682	0.1599	0.1100
VRSN	0.5551	0.3389	0.3199
VRTX	0.5007	0.2135	0.1679
WCRX	0.5106	0.3447	0.1688
WFMI	0.4544	0.2279	0.1042
YHOO	0.3750	0.2145	0.1299

We compute the cross-correlation coefficient $r(0)$ between query and trading volumes after removing the days characterized by the highest trading volumes, respectively the top five and top ten events are removed. A significant correlation is still observed for most of the stocks considered.
