# Supplementary material for: Web Search Queries Can Predict Stock Market Volumes
Source: PLoS One. 2012 Jul 19;7(7):e40014. doi: 10.1371/journal.pone.0040014 (PMC3400625; doi:10.1371/journal.pone.0040014)
Supplement: Supporting Information S2 — Detailed results of the three tests proposed, beyond Granger test, to validate the finding that query volumes anticipate trading volumes. (PDF) [file pone.0040014.s002.pdf]

SUPPORTING INFORMATION S2: Web search queries can predict stock market volumes.

Ilaria Bordino[1], Stefano Battiston[2], Guido Caldarelli[3,4,5], Matthieu Cristelli[3,*], Antti Ukkonen[1], Ingmar Weber[1]
1 Yahoo! Research, Barcelona, Spain
2 ETH Chair of System Design, Zurich, Switzerland
3 Inst. of Complex Systems CNR, "Sapienza" University, Rome, Italy
4 London Institute for Mathematical Sciences, London, United Kingdom
5 IMT - Institute for Advanced Studies, Lucca, Italy
∗ E-mail: matthieu.cristelli@roma1.infn.it

1 Beyond Granger Tests: Tables

Table S 1. Test 1: p-values for the 26 companies for which $Q \to V$ at $p = 0.01$

Ticker	$p - val(Q \to V)$	$p - val(V \to Q)$	CCF
**atvi**	0.000100	0.995100	0.39
**csco**	0.000100	1.000000	0.74
**expe**	0.000100	0.997100	0.52
**ilmn**	0.000100	0.996800	0.40
**isrg**	0.000100	0.998900	0.67
**nflx**	0.000100	1.000000	0.68
**nvda**	0.000100	1.000000	0.79
**rimm**	0.000100	1.000000	0.66
**altr**	0.000200	0.999900	0.55
**msft**	0.000200	0.961500	0.42
**symc**	0.000200	0.986800	0.58
**mrvl**	0.000500	0.990800	0.40
**orcl**	0.000500	0.966600	0.52
**erts**	0.000800	0.954600	0.62
**amgn**	0.000900	0.949500	0.36
**ffiv**	0.001400	0.793400	0.65
**ntap**	0.001800	0.968100	0.61
**bbby**	0.001900	0.983300	0.43
**apol**	0.002000	0.896500	0.79
**amzn**	0.002100	0.769600	0.48
**urbn**	0.002700	0.880500	0.37
**vrtx**	0.003200	0.973900	0.50
**adbe**	0.004300	0.766700	0.85
**qgen**	0.006000	0.981300	0.35
**chrw**	0.007300	0.965600	0.16
**stx**	0.008600	0.995600	0.37

Table S 2. Test 1: p-values for the ten companies with smallest $p - val(V \rightarrow Q$

**Ticker**	$\mathbf{p - val(Q \rightarrow V)}$	$\mathbf{p - val(V \rightarrow Q)}$
**dltr**	0.957000	0.019700
**mxim**	0.963700	0.060700
**lltc**	0.936800	0.106900
**rost**	0.851700	0.142600
**cmcsa**	0.913600	0.175900
**vrsn**	0.781100	0.176800
**infy**	0.750700	0.183600
**flir**	0.836400	0.193800
**vmed**	0.805600	0.220700
**intu**	0.821200	0.270100

Table S 3. List of tickers for which Test 2 gives significant results

**Ticker**	**Outcome**
aapl	$Q \to V$
adbe	$Q \to V$
adp	$Q \to V$
akam	$Q \to V$
altr	$Q \to V$
amgn	$Q \to V$
amzn	$Q \to V$
apol	$Q \to V$
atvi	$Q \to V$
bbby	$Q \to V$
bidu	$Q \to V$
biib	$Q \to V$
brcm	$Q \to V$
celg	$Q \to V$
ceph	$Q \to V$
csco	$Q \to V$
ctrp	$Q \to V$
ctxs	$Q \to V$
dltr	$Q \to V$
erts	$Q \to V$
esrx	$Q \to V$
expd	$Q \to V$
expe	$Q \to V$
ffiv	$Q \to V$
fslr	$Q \to V$
gild	$Q \to V$
grmn	$Q \to V$
ilmn	$Q \to V$
infy	$Q \to V$
intc	$Q \to V$
iisrg	$Q \to V$
klac	$Q \to V$
lrcx	$Q \to V$
mchp	$Q \to V$
micc	$Q \to V$
mrvl	$Q \to V$
msft	$Q \to V$
nflx	$Q \to V$
nihd	$Q \to V$
ntap	$Q \to V$
nvda	$Q \to V$
orcl	$Q \to V$
pcar	$Q \to V$
pcln	$Q \to V$
rimm	$Q \to V$
sbux	$Q \to V$
shld	$Q \to V$
sndk	$Q \to V$
srcl	$Q \to V$
stx	$Q \to V$
symc	$Q \to V$
urbn	$Q \to V$
wcrx	$Q \to V$
wfmi	$Q \to V$
yhoo	$Q \to V$

Table S 4. List of tickers for which Test 2 gives significant results

**Ticker**	**Outcome**
joyg	$V \to Q$
lltc	$V \to Q$
rost	$V \to Q$
teva	$V \to Q$
vrsn	$V \to Q$
vrtx	$V \to Q$

Table S 5. Outcome of Test 3

Ticker	p − val(Q → V)	p − val(V → Q)	Ticker	p − val(Q → V)	p − val(V → Q)
aapl	0.000002	0.006796	joyg	0.000000	0.000000
adbe	0.000000	0.000000	klac	0.000000	0.000000
adp	0.000000	0.000237	linta	0.030768	0.000000
adsk	0.000000	0.000000	lltc	0.000000	0.000000
akam	0.000000	0.000000	lrcx	0.000000	0.000000
altr	0.000000	0.000000	mat	0.000000	0.000970
amat	0.000000	0.000151	mchp	0.000000	0.000000
amgn	0.000000	0.000545	micc	0.000000	0.000000
amzn	0.000000	0.000000	mrvl	0.000000	0.000000
apol	0.000000	0.000000	msft	0.000000	0.005801
atvi	0.000000	0.000125	mu	0.000002	0.048229
bbby	0.000000	0.000061	mxim	0.000000	0.000000
bidu	0.000000	0.000000	myl	0.000000	0.000000
biib	0.000000	0.000229	nflx	0.000000	0.000003
bmc	0.000000	0.000000	nihd	0.000000	0.000000
brcm	0.000000	0.000000	ntap	0.000000	0.000004
celg	0.000575	0.006149	nvda	0.000000	0.000001
ceph	0.000000	0.000000	nwsa	0.000000	0.000000
chkp	0.033902	0.000000	orcl	0.000000	0.000000
chrw	0.000000	0.000000	payx	0.000027	0.000000
cmcsa	0.000000	0.000000	pcar	0.000000	0.000000
csco	0.000000	0.000000	pcln	0.000000	0.000000
ctrp	0.000000	0.000000	qcom	0.000000	0.000000
ctsh	0.000000	0.000005	qgen	0.000000	0.000000
ctxs	0.000000	0.004119	rimm	0.000000	0.000000
dltr	0.000000	0.000000	rost	0.000000	0.000000
dtv	0.000001	0.000001	sbux	0.000000	0.000044
erts	0.000000	0.000000	shld	0.000000	0.000000
esrx	0.000000	0.000000	sial	0.000080	0.024980
expd	0.000000	0.000000	sndk	0.000000	0.000000
expe	0.000000	0.000449	spls	0.000006	0.000000
ffiv	0.000000	0.000000	srcl	0.000000	0.000000
fisv	0.054118	0.000000	stx	0.000000	0.000000
flir	0.000005	0.000000	symc	0.000000	0.000001
fslr	0.000000	0.000000	teva	0.000000	0.000567
gild	0.000000	0.000000	urbn	0.000000	0.000000
goog	0.000000	0.155566	vmed	0.000003	0.000000
grmn	0.000000	0.000000	vod	0.000000	0.004847
hsic	0.000000	0.000000	vrsn	0.000000	0.000000
ilmn	0.000000	0.001255	vrtx	0.000000	0.057984
infy	0.000000	0.000000	wcrx	0.000000	0.000000
intc	0.000000	0.000000	wfmi	0.000000	0.000000
intu	0.000000	0.000000	yhoo	0.000000	0.000000
isrg	0.000000	0.026642			
